# Saikosaponin A induces cellular senescence in triple-negative breast cancer by inhibiting the PI3K/Akt signalling pathway

**DOI:** 10.3389/fphar.2025.1532579

**Published:** 2025-04-25

**Authors:** Yingchao Wu, Liushan Chen, Dajin Pi, Jiaqi Cui, Yuqi Liang, Peng Wu, Mingzi Ouyang, Qian Zuo

**Affiliations:** ^1^ State Key Laboratory of Traditional Chinese Medicine Syndrome, The Second Affiliated Hospital of Guangzhou University of Chinese Medicine, Guangzhou, Guangdong, China; ^2^ The Second Clinical College of Guangzhou University of Chinese Medicine, Guangzhou, Guangdong, China; ^3^ Department of Breast, Guangdong Provincial Hospital of Chinese Medicine, Guangzhou, Guangdong, China; ^4^ Guangdong Academy of Traditional Chinese Medicine, Guangzhou, Guangdong, China; ^5^ Guangdong Provincial Key Laboratory of Clinical Research on Traditional Chinese Medicine Syndrome, Guangzhou, Guangdong, China; ^6^ School of Traditional Chinese Medicine, Jinan University, Guangzhou, Guangdong, China

**Keywords:** saikosaponin a, breast cancer, network analysis, cellular senescence, PI3K/Akt signaling pathway

## Abstract

**Background:**

Breast cancer has now become the most prevalent cancer worldwide. Existing therapeutic agents are generally accompanied by significant side effects. Here, we highlight Saikosaponin A (SSA), a promising natural metabolite characterized by low toxicity, demonstrating significant efficacy against breast cancer through the induction of cellular senescence.

**Methods:**

The antitumor property of SSA was determined via MTT colorimetric assay, 5-ethynyl-2′-deoxyuridine (EdU) incorporation assay, colony formation, and propidium iodide (PI) staining *in vitro*, as well as xenograft *in vivo* model. A network approach was used to predict potential targets of SSA reevant for a potential anti-tumor effect and verified through senescence-associated β-galactosidase (SA-β-gal), flow-cytometry analysis, RT-PCR, Western blotting, and immuno-histochemistry assay.

**Results:**

SSA significantly suppressed proliferation and triggered cell cycle arrest of SUM159PT and MDA-MB-231 cells. Revealed by network analysis, cellular senescence, and phosphatidylinositol-3-kinase (PI3K)/Akt signaling pathway were implemented in the anti-tumor effects of SSA. SSA-stimulated senescence was associated with increased ROS production, distinct senescence-associated secretory phenotype (SASP), and restricted PI3K/Akt signaling, as well as p21 and p53 accumulation. Furthermore, SSA displayed inhibitory effects on tumor growth with minimal toxicity in animal studies, accompanied by activated biomarkers of cellular senescence and decreased expression of p-Akt and p-PI3K.

**Conclusion:**

Taken together, based on the preliminary results of network analysis and further experimental validation, this study revealed that SSA significantly induced cell cycle arrest and senescence, and the inhibition of ROS-mediated PI3K/Akt pathway may be the potential mechanism in this process.

## 1 Introduction

Breast cancer is now becoming the most common cancer worldwide (Arnold et al., 2022). As the most aggressive form, triple-negative breast cancer (TNBC) consists of approximately 10%–15% of the total cases of breast cancer. Owing to its significant proliferative activity, high growth rate, and limited treatment options, TNBC is in close relationship to a higher recurrence rate and a poorer prognosis in comparison with other subtypes of breast cancer (Zagami and Carey, 2022; Zhou and Chen, 2023). Consequently, there is an imperative need to explore and exploit therapeutic avenues for addressing TNBC. Natural products (NPs) stand as an invaluable wellspring of inspiration in the design and development of pharmaceuticals with limited side effects and less costs. Many literature provide the therapeutic properties of active metabolites from botanical drugs on breast cancer, via suppression of cancer cell proliferation and metastasis, or modulation of immune responses (Corso et al., 2021; Rampogu et al., 2022). Saikosaponin A (SSA), isolated from the botanical drug Radix Bupleurum, possesses multiple pharmacological activities. Prior studies have elucidated the therapeutic efficacy of SSA in breast cancer, highlighting its role in regulating antitumor immunity, cell apoptosis, migration, and invasion. However, the impact and mechanistic understanding of SSA on the cell cycle regulation in breast cancer remains largely unexplored.

Cellular senescence, characterized by an irreversible arrest in the cell cycle, has arisen as a therapeutic target for varied types of cancer (Rodier and Campisi, 2011). Moreover, exposure to various anti-tumor agents has been found to trigger premature senescence in cancer cells (Sieben et al., 2018). As a result, the recognition of cellular senescence as a potent strategy in cancer therapy is growing, attributed to the non-proliferative state induced in cells exposed to low concentrations of chemotherapeutic agents. Various factors can instigate cellular senescence, with the age-related accumulation of reactive oxygen species (ROS) emerging as a notable modulator. This accumulation interfaces with both inflammatory and nutrient-sensing pathways, collectively accelerating the advancement of the cellular senescence program (Davalli et al., 2016). In the current study, TNBC cells displayed a remarkable increase in ROS levels under SSA treatment. Additionally, network analysis was employed in SSA-treated TNBC cells. Pathway enrichment analyses indicated that cellular senescence might be partly responsible for the anti-tumor activities of SSA. However, the response targets of SSA in cellular senescence remain to be elucidated.

As an emerging discipline rooted in bioinformatics, high-throughput omics data analysis, online databases, and network analysis have been used to elucidate the potential targets for pharmacological activity of natural products. For example, by employing the network analysis and other *in silico* approaches, the natural metabolite Juglanthraquinone C has been identified for the first time as possessing anti-cancer properties, with a total of 31 potential therapeutic targets obtained (Qayoom et al., 2023). In this study, we performed a series of functional assays to investigate whether SSA regulates the ROS-mediated Phosphatidylinositol-3-Kinase (PI3K)/Akt signaling pathway to trigger cellular senescence of TNBC, ultimately resulting in significant cell cycle arrest. Importantly, we have confirmed for the first time that SSA inhibits breast cancer progression by inducing cell senescence, which has never been reported in previous studies.

## 2 Materials and methods

### 2.1 Cell culture and treatment

Human TNBC cell lines SUM159PT, MDA-MB-231,and human normal mammary epithelial cell MCF 10A were obtained from the China Center for Type Culture Collection and cultured in medium (HyClone, GE Life Sciences, Logan, UT, United States), added with 10% fetal bovine serum (Thermo Fisher Scientific, Waltham, MA, United States) under conditions of 37°C and 5% CO_2_. SSA (Yuanye Biotechnology, Shanghai, China) was freshly dissolved at 5 μM, 10 μM, and 15 μM before each experiment. For combination of SSA and N-acetylcysteine (NAC) treatment, both SUM159PT and MDA-MB-231 were exposed to 10 μM SSA for 48 h after 2 mM NAC pretreatment.

### 2.2 Cell viability assessment

Cells were exposed to SSA at indicated concentrations for 24 h, 48 h, and 72 h. Cell viabilities were evaluated via MTT assay (Thermo Fisher Scientific, Waltham, MA, United States). An automated microplate spectrophotometer (BioTek Instruments, Winooski, VT, United States) was used to collect the absorbance of each well at 490 nm.

### 2.3 EdU labeling assay

A density of 5 × 10^4^ SUM159PT and MDA-MB-231 cells were treated with varied concentrations of SSA (0–10 μM). Following a 48-h incubation period, the BeyoClick™ EdU Cell Proliferation Kit (Beyotime Biotechnology, Shanghai, China) was employed to quantify proliferating cells in *in vitro* cultures and tissue sections. Photomicrographs were captured immediately via an inverted microscope at 100× magnification (Nikon, Japan).

### 2.4 Colony formation assay

A density of 100 SUM159PT and MDA-MB-231 cells were exposed to SSA treatment for 14 days in 6-well plates. Subsequently, 75% ethanol and 1% crystal violet were used to fix and stain the colonies, respectively.

### 2.5 Network analysis

For screening for SSA-related targets, 7 SSA-related targets from the Traditional Chinese Medicine Systems Pharmacology Database (TCMSP) (https://old.tcmsp-e.com/tcmsp.php), 300 SSA-related targets from the Pharmmapper database (http://www.lilab-ecust.cn/pharmmapper/index.html), another 7 SSA-related targets from the Swiss Target Prediction database (http://www.swisstargetprediction.ch/), and 154 SSA-related targets from the Super-PRED database (https://prediction.charite.de/index.php?site=chemdoodle_search_target) were collected. All the collected targets were standardized via UniProt (https://www.uniprot.org). For screening for TNBC-related targets, 2640 TNBC-related targets from the Genecards database (https://www.genecards.org) and an additional 241 TNBC-related targets from the Online Mendelian Inheritance in Man (OMIM) database (https://omim.org) were standardized using the DAVID database (https://david.ncifcrf.gov/home.jsp). For protein-protein interaction (PPI) network analysis and screening of core targets of SSA, the String database (https://cn.string-db.org) was employed to analyze the PPI network for the targets of SSA in TNBC with the organism specified as “*Homo sapiens*” and a confidence score threshold of ≥0.4. Subsequently, Cytoscape software (Version: 3.9.0) was employed to construct a “metabolite-target-disease” network, facilitating the identification of core targets and hub genes associated with SSA in TNBC. For Gene ontology (GO), Kyoto Encyclopedia of genes and genomes (KEGG) pathway, Disease Ontology (DO), and Reactome pathway enrichment analysis, pathway enrichment analysis of SSA targets was carried out utilizing the Xiantao Academic online platform (https://www.xiantaozi.com). The outcomes of the enrichment analysis were then visualized using the NovoMagic online platform (https://magic.novogene.com).

### 2.6 Molecular docking

The binding affinity between SSA and the core targets was validated by molecular docking. The 2D structure of SSA retrieved from the PubChem database was imported into ChemDraw 3D (Version 19.0). The MM2 module was employed for energy minimization to obtain the most energetically favorable conformation. Protein structures, acquired from the RCSB PDB database (https://www.rcsb.org), served as receptors. Because both drug targets and disease targets are derived from human databases, we use human (*Homo sapiens*) proteins for molecular docking. These structures were visualized separately using PyMOL, and subjected to dehydration, hydrogenation, and charge calculations through Mgtools (Version 1.5.6). Ligand and receptor structures were saved as pdbqt files. Since STAT3, BCL2, AKT1, ESR1, HSP90AA1, and HSP90AB1 proteins contain specific ligands, we used the target pockets of specific ligands for docking. Since MYC and NFKB1 proteins do not contain specific ligands, we first generate docking pockets based on the overall structure of proteins, and then perform molecular docking based on the generated docking pockets. Subsequent molecular docking was performed utilizing AutoDock Vina (version 1.5.6), and the resulting higher-scoring conformations were visualized with PyMOL. The PLIP web tool (https://plip-tool.biotec.tu-dresden.de/plip-web/plip/index) facilitated the visualization of docking points, while the TCMNPAS online platform (Version 1.0; http://54.223.75.62:3838/npa) was utilized for calculating the Root Mean Square Deviation (RMSD) of the docking process.

### 2.7 Cellular thermal shift assay (CETSA)

For SUM159PT cells, they were lysed by repeatedly being frozen and thawed with liquid nitrogen for 3 times. The cell lysates were divided into two aliquot, one as the control and the other treated with SSA (15 μM) for 30 min at room temperature. Then, the lysates were heated at the desired temperatures (57°C–73°C) and cooled on ice. The protein bands were detected with Immunoblotting.

### 2.8 Bioinformatics analysis

The Xiantao Academic online platform was employed for visualizing the mRNA expression of core genes in breast tissues based on data retrieved from The Cancer Genome Atlas (TCGA) database (https://portal.gdc.cancer.gov). Protein expressions of core targets in breast tissues were sourced from the Human Protein Atlas (HPA) database (https://www.proteinatlas.org). To illustrate the association of core targets with the prognosis of breast cancer, the Kaplan-Meier Plotter database (http://kmplot.com/analysis/index.php?p=background) was utilized to generate survival curves.

### 2.9 Assessment of senescence-associated β-galactosidase (SA-β-gal) activity

Cells were fixed and exposed to freshly prepared SA-β-gal staining according to the manufacturer’s instructions (Beyotime Biotechnology, Shanghai, China). Total cell numbers and SA-β-gal-positive cell numbers were randomly counted across 5–10 fields per slide/well. The percentage of SA-β-gal-positive cells was calculated based on the positive cells per area.

### 2.10 Flow cytometric analysis

Cell cycle arrest was conducted using propidium iodide staining (Beyotime Biotechnology, Shanghai, China) and analyzed by a BD FACSCelesta flow cytometer (BD Biosciences, San Diego, CA). To measure intracellular ROS levels, cells stained with 10 µM dichloro-dihydro-fluorescein diacetate (DCFH-DA) dye were cultured in serum-free culture medium at 37°C for 20 min, and analyzed by a BD FACSCelesta flow cytometer after washing with PBS.

### 2.11 Reverse transcription-PCR

Total RNA was extracted 48 h post-SSA treatment by using the RNeasy Mini kit (74,104, Qiagen). The extracted RNA was utilized for reverse transcription following the manufacturer’s instructions. QuantiFast Probe RT-PCR Kit (204,443, Qiagen) was used to perform real-time PCR product quantification. The housekeeping gene GAPDH served as the internal control for RNA integrity and expression normalization. Primer sequences are summarized in Supplementary Table S1.

### 2.12 Immunoblotting

After treatment as indicated, whole-cell lysates were prepared using RIPA buffer for the Western blot assay. Blots were incubated with primary antibodies targeting p53 and GAPDH (Proteintech, Rosemont, IL, United States), and PI3K, Akt, phospho-Akt (Ser124), p21 Cip1 (Signalway Antibody, Greenbelt, Maryland, United States) overnight at 4°C, respectively. This was followed by incubation with peroxidase-conjugated appropriate secondary antibodies. Immunocomplexes were visualized using chemiluminescence (Millipore).

### 2.13 Cell and tissue heterochromatin protein 1 (HP1) concentration detection

Human HP1 ELISA kit (Cat. #AE96433Hu) was purchased from MAIGE (Jiangsu, China). HP1 levels in cell and tissue were determined by ELISA according to the manufacturer’s instructions.

### 2.14 Nude mice xenograft model

The animal study was approved by the Ethics Committee for Animal Experiments of Jinan University (Approval No. IACUC-20230507–07). Tumor-bearing mice were established by subcutaneous injection of 5 × 10^6^ SUM159PT or MDA-MB-231 cells suspended in a 1: 1 mixture of PBS: Matrigel (Corning Incorporated, Tewksbury, MA, United States). SSA was administered daily via intraperitoneal injection (25 mg/kg/g), starting on day 5 after cancer cell inoculation and continuing for a total of 28 days. At the end of this animal study, sections of each tumor from different groups were collected, frozen, and paraffin-sectioned for senescence-associated β-galactosidase staining. Immunohistochemical analyses of p16, p21, p53, and Ki67 were also performed. ImageJ was used to quantify positive cells in IHC, where the “Threshold” range for positive cells was 33–195. Hearts, kidneys, and lungs were collected for histologic analyses.

### 2.15 Statistical analysis

All *in vitro* experiments were conducted in triplicate. Statistical analyses, including two-tailed Student’s tests, one-way ANOVA, two-way ANOVA, and the Chi-square test, were performed using GraphPad Prism software v.5.01 (San Diego, CA, United States). The results were presented as means ± SD, and significance was set at *P* < 0.05.

## 3 Results

### 3.1 SSA inhibits the growth of TNBC cells *in vitro* and *in vivo*

**FIGURE 1:**
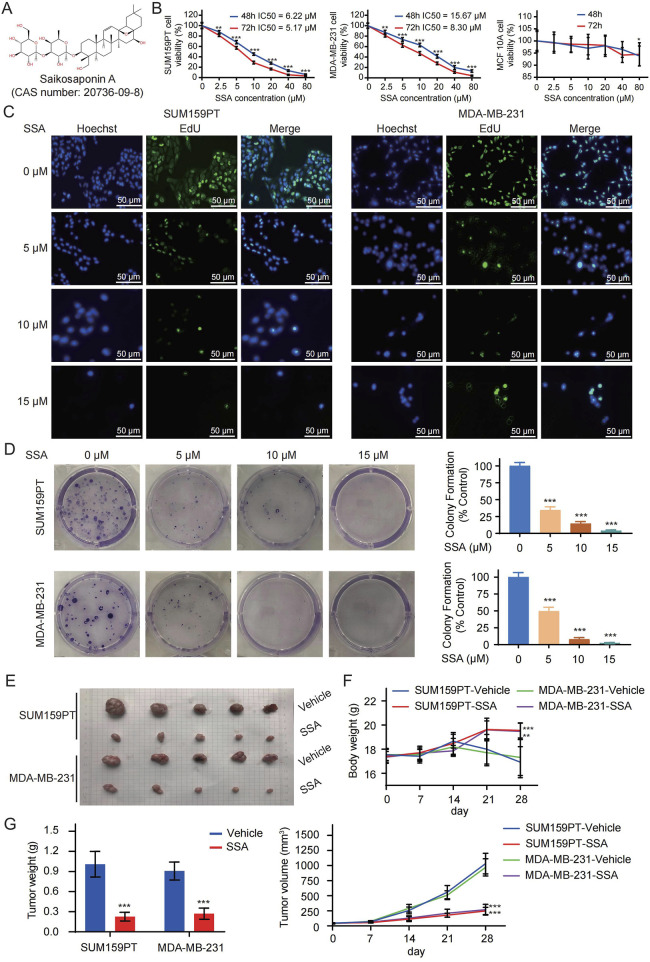
SSA inhibits the growth of TNBC cells *in vitro* and *in vivo*. **(A)** The chemical structure of SSA (CAS number: 20736-09-8). **(B)** The viability of SUM159PT, MDA-MB-231, and MCF 10A cells treated with different concentrations of SSA in medium for 48 and 72 h **(C)** EdU labelling and colony formation assays **(D)** were used to evaluate the effect of different concentrations of SSA on cell proliferation. Nude mice bearing SUM159PT/MDA-MB-231-derived tumor xenografts were administered daily via intraperitoneal injection (25 mg/kg/g) or vehicle every 2 days. **(E)** Tumor image of the tumors. **(F)** The body weights of the nude mice during the experimental period. **(G)** Tumor growth curves and tumor weights showing that SSA exerted a significant inhibitory effect on the growth of the SUM159PT/MDA-MB-231-derived tumor xenografts (n = 6). All the data are presented as the means ± SD. ^*^ *P* < 0.05, ^**^ *P* < 0.01, ^***^ *P* < 0.001.

The chemical structural formula of SSA is shown in Figure 1A. As illustrated in Figure 1B, after 48 h of treatment, the IC50 values for inhibiting the proliferation of SUM159PT and MDA-MB-231 cells were 6.22 μM and 15.67 μM, respectively, but SSA had no significant effect on normal human mammary epithelial cells MCF 10A at this concentration. Subsequently, doses of 5 μM–15 μM SSA were selected to treat SUM159PT and MDA-MB-231 cells. EdU labeling, together with colony formation assays, was employed to detect the effects of SSA on the proliferation and clonality of TNBC cells. The results, presented in Figures 1C,D, reveal that SSA at doses of 0 μM–15 μM significantly impeded the viability, proliferation, and clonality of SUM159PT and MDA-MB-231 cells.

Drawing from the observations in SSA-treated TNBC cells, we proceeded to evaluate the alteration of TNBC cell growth *in vivo* under SSA treatment. As illustrated in Figure 1F, mice subjected to SSA treatment exhibited a relatively modest reduction in body weight compared to those receiving the vehicle control. Following a 28-day course of SSA treatment, both tumor volumes and weights exhibited significant reductions in SSA-treated mice (Figures 1E,G). To further investigate proliferation indices, EdU labeling, and Ki-67 immunohistochemical analysis were conducted. The xenografts from SSA-treated mice displayed a notable decrease in the cell proliferation rate (Supplementary Figures S1A,S1B). Collectively, these data suggested its potential as an anticancer agent.

### 3.2 The identified targets of SSA for TNBC treatment and the involved biological terms and pathways

**FIGURE 2:**
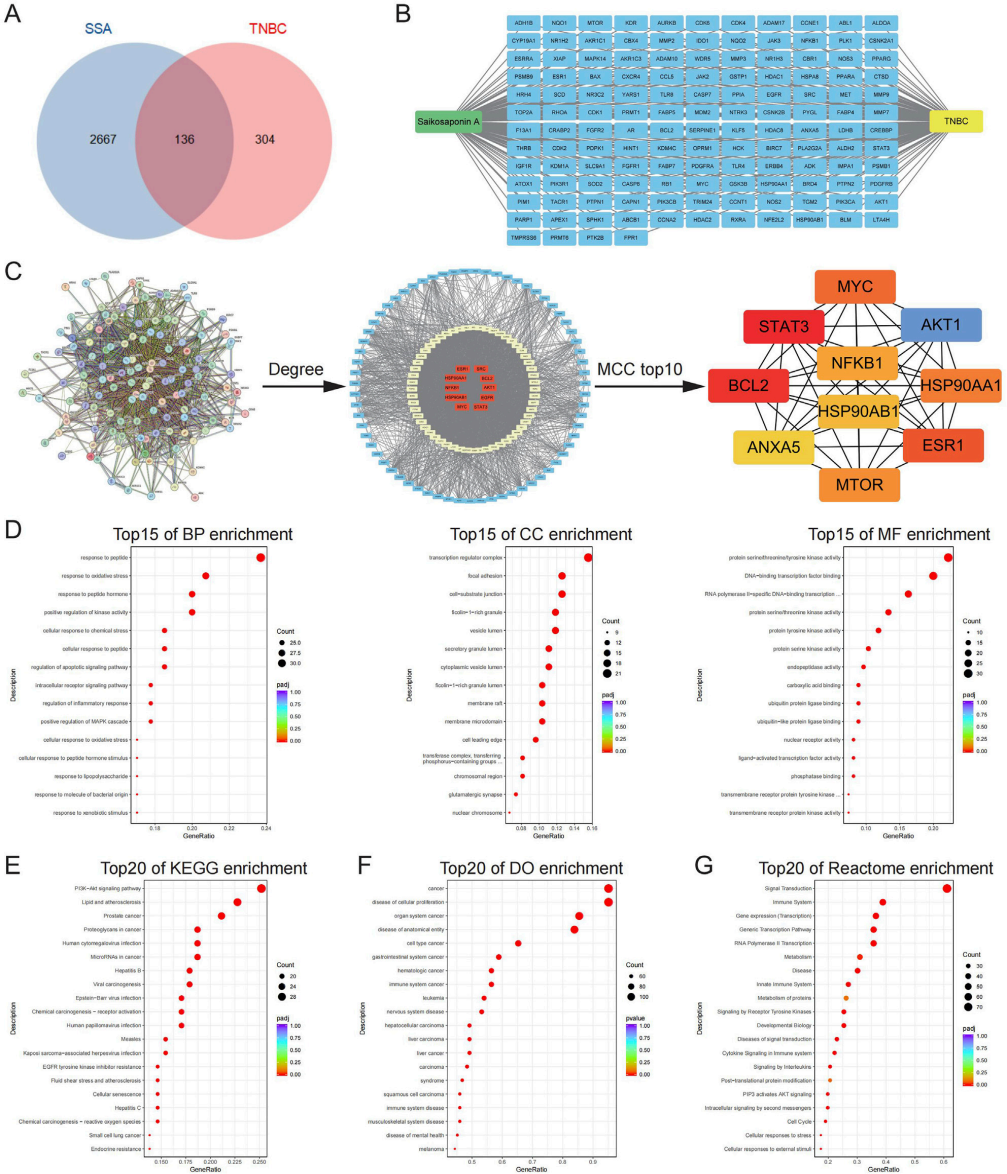
The identified targets of SSA for TNBC treatment and the involved biological terms and pathways. **(A)** A total of 136 SSA targets for TNBC were screened by Venn diagram. **(B)** Targets of SSA for TNBC. **(C)** PPI network analysis of SSA targets for TNBC was performed by using the String database. The red nodes in the middle represented the larger degree values and small networks are the top 10 targets obtained by the MCC algorithm. **(D)** The top 15 significant enriched terms by GO functional enrichment analysis were shown at BP level, CC level and MF level. **(E–G)** The top 20 significant KEGG, DO, and Reactome enriched terms.

By consolidating data and predictions from the TCMSP, PharmMapper, Swiss Target Prediction, and Super-PRED databases, a total of 440 SSA-related targets were identified. Additionally, 2803 TNBC-related targets were obtained from the Genecards and OMIM databases. The intersection of SSA and TNBC targets was then determined using a Venn diagram approach, pinpointing 136 specific targets of SSA for TNBC (Figures 2A,B). Subsequently, the PPI network analysis of these 136 targets relying on the String database is shown in Figure 2C. In the graphical representation, the red nodes at the center represent targets with higher degree values, while the cyan nodes on the periphery indicate those with smaller degree values. The Maximal Clique Centrality (MCC) topology algorithm was employed to identify the top 10 ranked nodes. Targets with larger degree values and those ranking in the top 10 according to the MCC topology algorithm are likely to be hub genes, potentially representing core targets of SSA treatment in TNBC. These include genes such as STAT3, TNBCL2, AKT1, ESR1, MYC, HSP90AA1, NFKB1, and HSP90AB1.

The identified targets of SSA for TNBC were associated with a comprehensive array of biological terms and pathways. Specifically, there were 2,217 associated terms at the Biological Process (BP) level, 6 at the Cellular Component (CC) level, and 94 at the Molecular Function (MF) level. The top 15 significant terms for each level are respectively depicted in Figure 2D. GO enrichment results suggested that specific targets were enriched in the response to oxidative stress, positive regulation of kinase activity, and protein serine/threonine/tyrosine kinase activity. Furthermore, the enrichment analysis encompassing KEGG pathways, DO, and Reactome pathways revealed extensive associations. There were 145 pathways identified in the KEGG analysis, 979 pathways in the DO analysis, and 3,565 pathways in the Reactome analysis, all related to the SSA targets for TNBC. The top 20 significant pathways from each of these analyses (KEGG, DO, and Reactome) are exhibited in Figures 2E–G, respectively. KEGG pathway enrichment results suggested that specific targets were enriched in the PI3K−Akt signaling pathway and cellular senescence. DO enrichment results suggested that specific targets were enriched in the disease of cellular proliferation and cell type cancer. Reactome pathway enrichment results suggested that specific targets were enriched in the signal transduction, signaling by receptor tyrosine kinases, PIP3 activates AKT signaling, intracellular signaling by second messengers and cell cycle. These results illustrate the broad and complex interactions and potential targets of SSA in the context of TNBC.

### 3.3 Analysis of SSA binding to core targets and relationship of core targets with the top15 GO and top20 KEGG, DO, and reactome pathways

**FIGURE 3:**
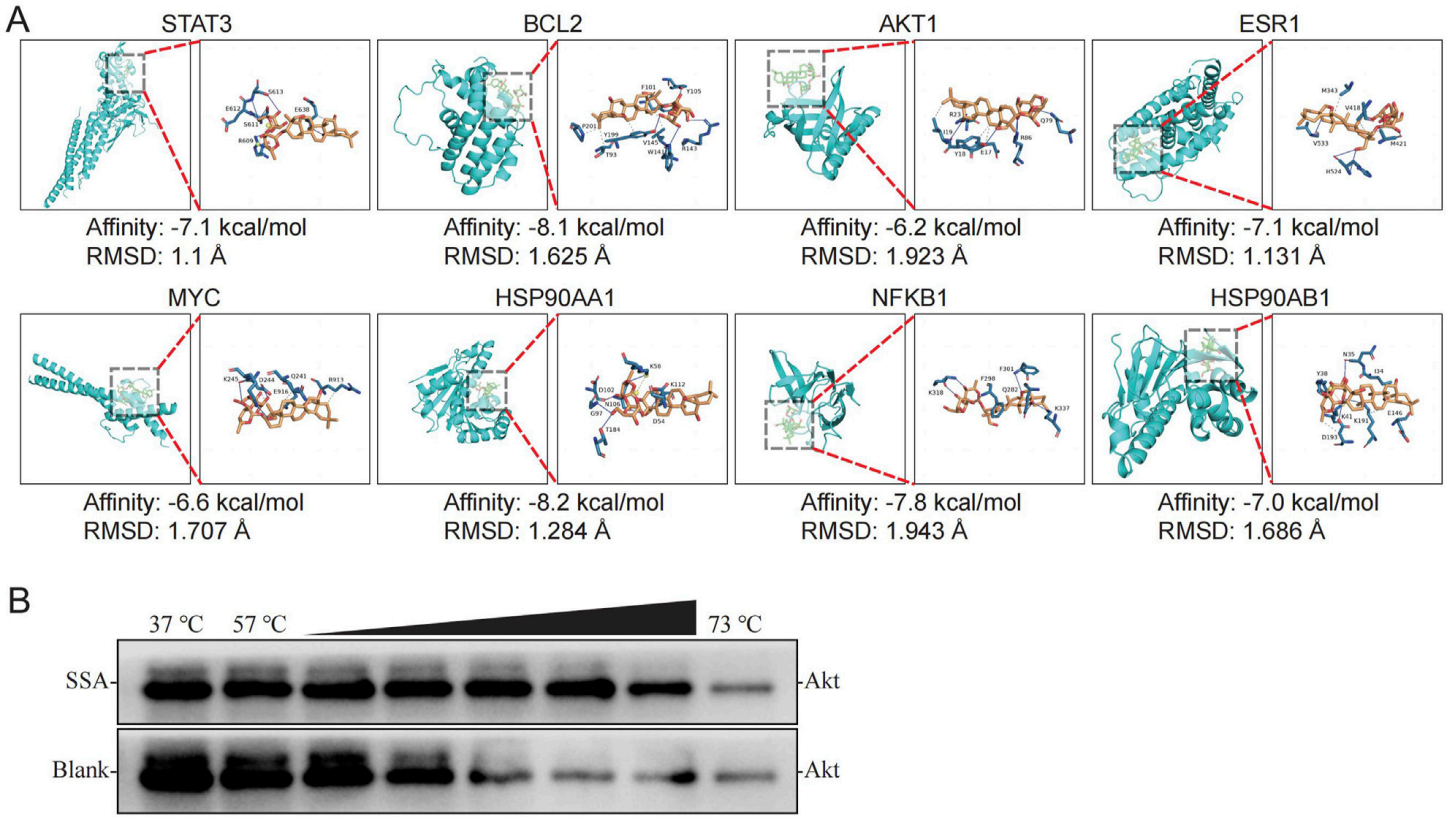
Analysis of SSA binding to core targets. **(A)** Molecular docking and binding energies of SSA and STAT3, TNBCL2, AKT1, ESR1, MYC, HSP90AA1,NFKB1, and, HSP90AB1, respectively. **(B)** CETSA results.

To verify the core targets of SSA treatment in TNBC, molecular docking was performed to evaluate the binding energy and activity between SSA and the indicated proteins, including STAT3 (6NJS), TNBCL2 (1YSW), AKT1 (1UNQ), ESR1 (3OS9), MYC (6G6J), HSP90AA1 (1OSF), NFKB (1NFI), and HSP90AB1 (1UYM). As illustrated in Figure 3A, the binding energies between SSA and these core targets were all less than −5.0 kcal/mol, and the Root Mean Square Deviations (RMSD) were all below 2. These values suggest a strong and stable binding affinity between SSA and the core targets, indicating effective molecular interactions.

In addition, the study explored how these core targets relate to the top enrichment terms derived from various analyses. Supplementary Figures S2A–S2D displayed the relationship of these core targets with the top 15 GO terms, the top 20 KEGG pathways, the top 20 DO terms, and the top 20 Reactome pathways, respectively. This comprehensive analysis revealed that each of the core targets is involved in these top enrichment terms, suggesting their broad and significant roles in the biological processes, pathways, and diseases related to TNBC. This integrative approach underscores the potential of SSA in targeting key molecular players in TNBC, providing valuable insights into its therapeutic targets. In addition, since the PI3K-Akt signaling pathway was significantly enriched in the enrichment analysis, and AKT1 was a core target, in order to avoid false positive results, CEAST was used to verify the affinity between SSA and Akt. The results showed that SSA treatment effectively protected Akt protein from temperature-dependent degeneration, indicating that SSA has a good affinity with Akt (Figure 3B).

### 3.4 Expression and survival analysis of the core targets of SSA

**FIGURE 4:**
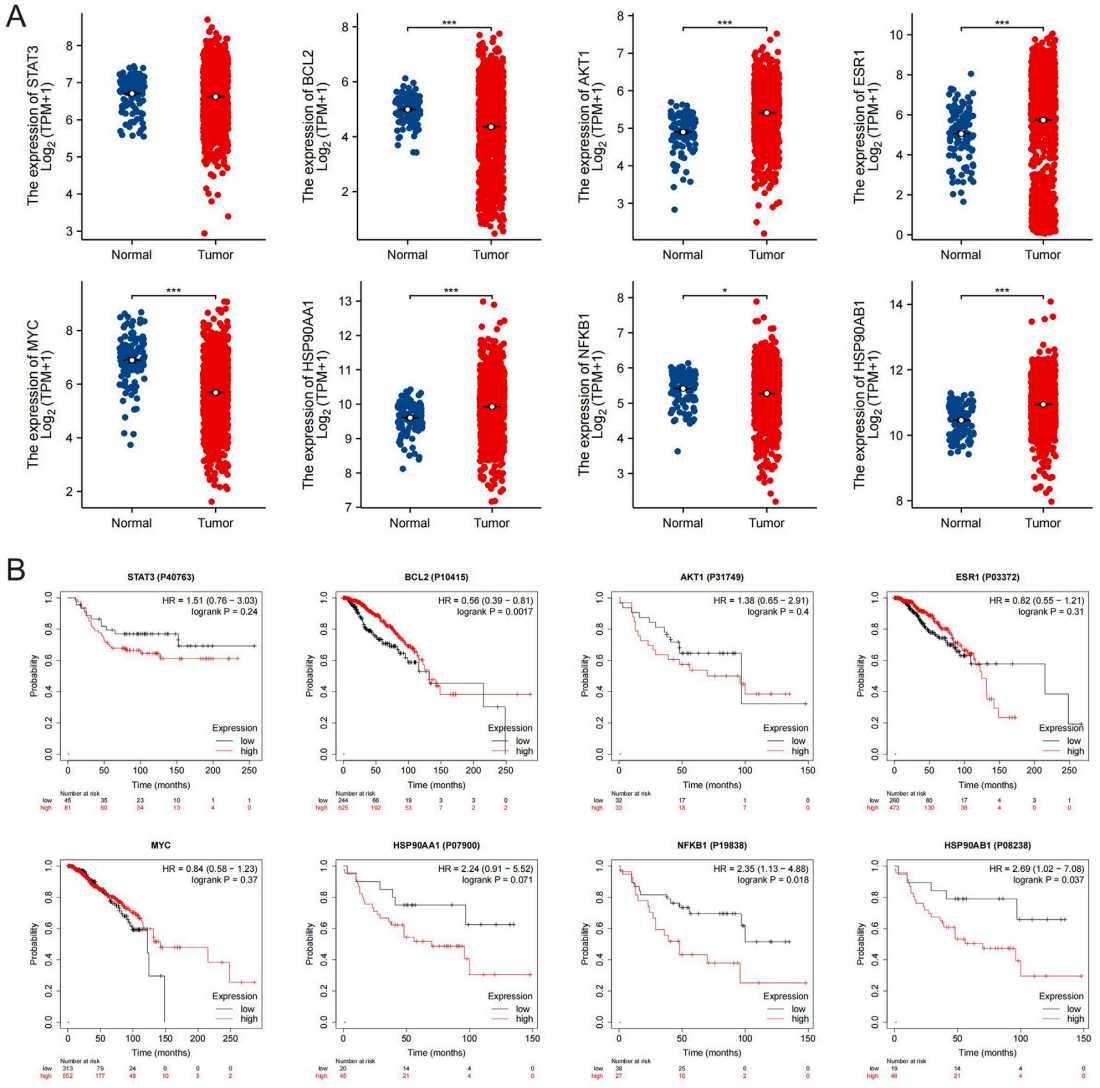
Expression and survival analysis of the core targets of SSA. **(A)** The mRNA expression level of the 8 core targets in TCGA breast cancer dataset; ^*^ *P* < 0.05, ^***^ *P* < 0.001. **(B)** Survival curves of the 8 core targets in the breast cancer protein dataset from the Kaplan-Meier Poltter.

Validation of the core targets of SSA in the TCGA database via GEPIA demonstrated that the mRNA expression of BLC2, AKT1, ESR1, MYC, HSP90AA1, NFKB1, and HSP90AB1 were significantly higher in breast cancer tissues (Figure 4A). Furthermore, no significant difference in STAT3 mRNA expression was observed. Additionally, the Human Protein Atlas database was employed to determine the differential protein expressions between BC and normal breast tissues. As is shown in Supplementary Figure S3, AKT1, ESR1, HSP90AA1, and HSP90AB1 were moderately expressed in normal breast tissues and highly expressed in BC tissues, whereas BCL2, MYC, and NFKB1 were obviously expressed in normal breast tissues. The protein expression of STAT3 in both tissues exhibits no significant difference. In short, the protein levels of the core targets of SSA were consistent with the results of differential analyses of mRNA levels.

The Kaplan-Meier survival analysis, as depicted in Figure 4B, explored the relationship between the expression of the core targets of SSA and the overall survival outcomes of breast cancer patients. The results indicate that patients with limited expression levels of HSP90AB1 and NFKB1 manifested markedly higher overall survival rates than those with elevated expression (*P* < 0.05). Conversely, patients with overexpression of BCL2 genes exhibited markedly better survival rates in comparison to those with lower expression levels of these genes (*P* < 0.05). Notably, the expression of STAT3, AKT1, ESR1, MYC, and HSP90AA1 genes did not display a statistically significant correlation with patient overall survival (*P* > 0.05).

### 3.5 SSA triggers ROS-mediated cell senescence in TNBC

**FIGURE 5:**
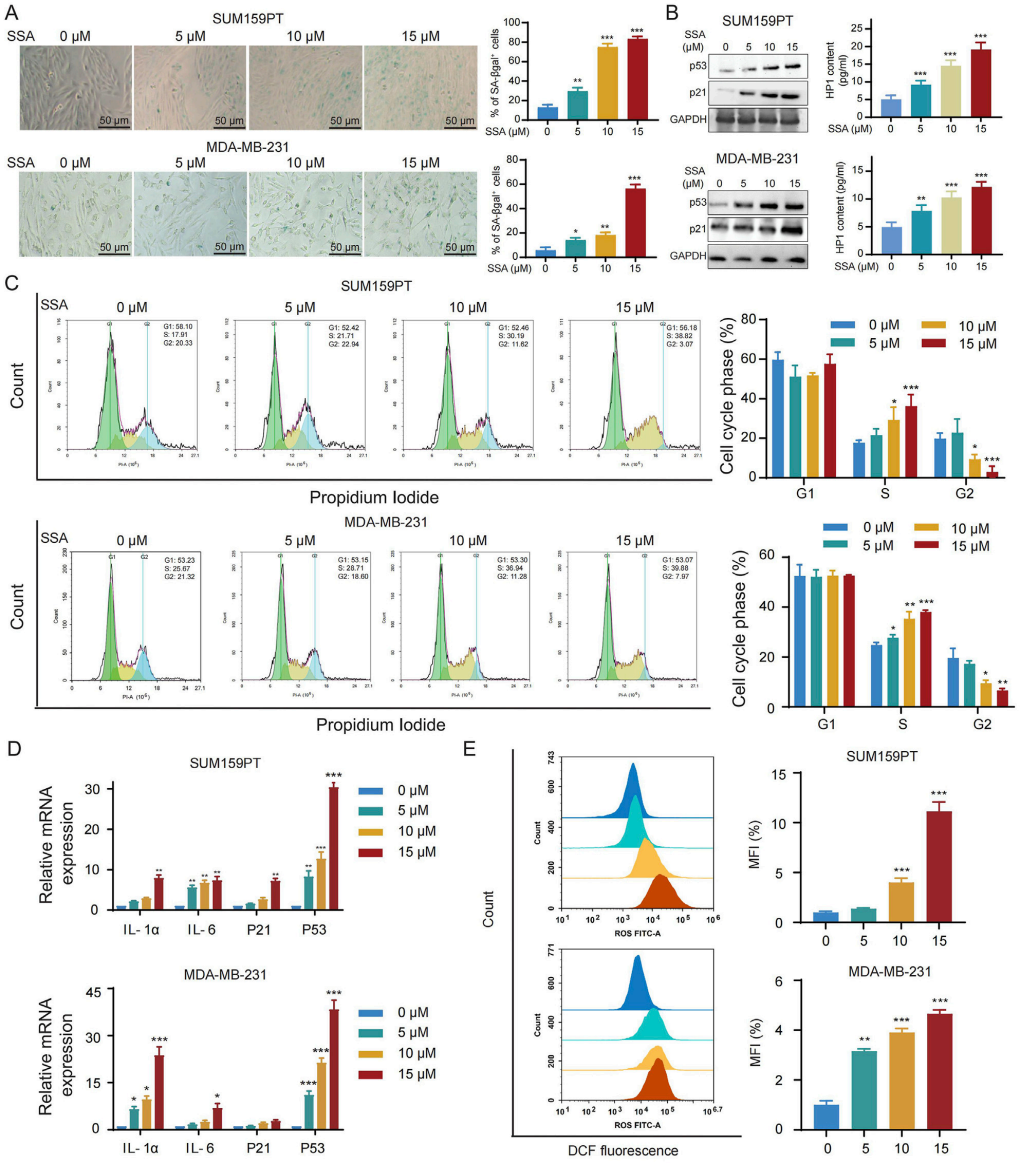
SSA triggers ROS-mediated cell senescence in TNBC. **(A)** Cellular senescence was detected by SA-β-gal staining in the absence or presence of the indicated concentrations of SSA for 48 h (n = 3). **(B)** Immunoblot analysis and ELISA of p53, p21, and HP1 in cells treated with vehicle or SSA for 48 h (n = 3). **(C)** Cell cycle was analyzed by PI staining in the absence or presence of the indicated concentrations of SSA for 48 h (n = 3). **(D)** Quantification for mRNA levels of SASP-related genes in SSA-treated cells which were determined by qRT-PCR (n = 3). **(E)** The accumulation of intracellular ROS was analyzed by flowcytometry in the absence or presence of the indicated concentrations of SSA for 48 h (n = 3). All the data are presented as the means ± SD. ^*^ *P* < 0.05, ^**^ *P* < 0.01, ^***^ *P* < 0.001.

Based on enrichment analysis in SSA-targeting datasets, cellular senescence serves a crucial role in the observed toxicities of SSA. We thus investigated whether cellular senescence is modulated by SSA in TNBC cells. Firstly, SA-β-Gal staining was utilized to evaluate the ability of SSA to trigger senescence in TNBC cells. As shown in Figures 5A,B, SSA treatment at 5 μM–15 μM significantly amplified the positive percentages of SA-β-gal-staining cells in a dose-dependent manner, which was consistent with the overexpression of senescence markers (p21, p53, and HP1). Furthermore, the regulatory role of SSA on cellular senescence was further validated by flow cytometry analysis of cell cycle. As shown in Figure 5C, a progressive increase was observed in the subG2-phase of TNBC cells and decrease in the subS-phase of TNBC under SSA treatment in both TNBC cells. Accompanied by cellular senescence, the senescence-associated secretory phenotype (SASP) is a key feature of senescent cells that comprises the release of secreted inflammatory cytokines (IL-1, IL-6), as well as the biomarkers of senescence (P53 and P21). Consistent with the previous findings, SSA significantly upregulated the expression of SASP-related genes (Figure 5D).

Next, we sought to investigate the mechanism by which SSA induced senescence in breast cancer cells. Cellular senescence can be activated by an array of factors, yet the production of ROS emerges as a key regulator in the acceleration of the cellular senescence program via inflammatory and nutrient-sensing pathways. As shown in Figure 5E, an obvious increase in ROS levels was detected in SSA-treated SUM159PT and MDA-MB-231 cells.

### 3.6 Activated ROS generation mediated the pharmacological activities of SSA in TNBC

**FIGURE 6:**
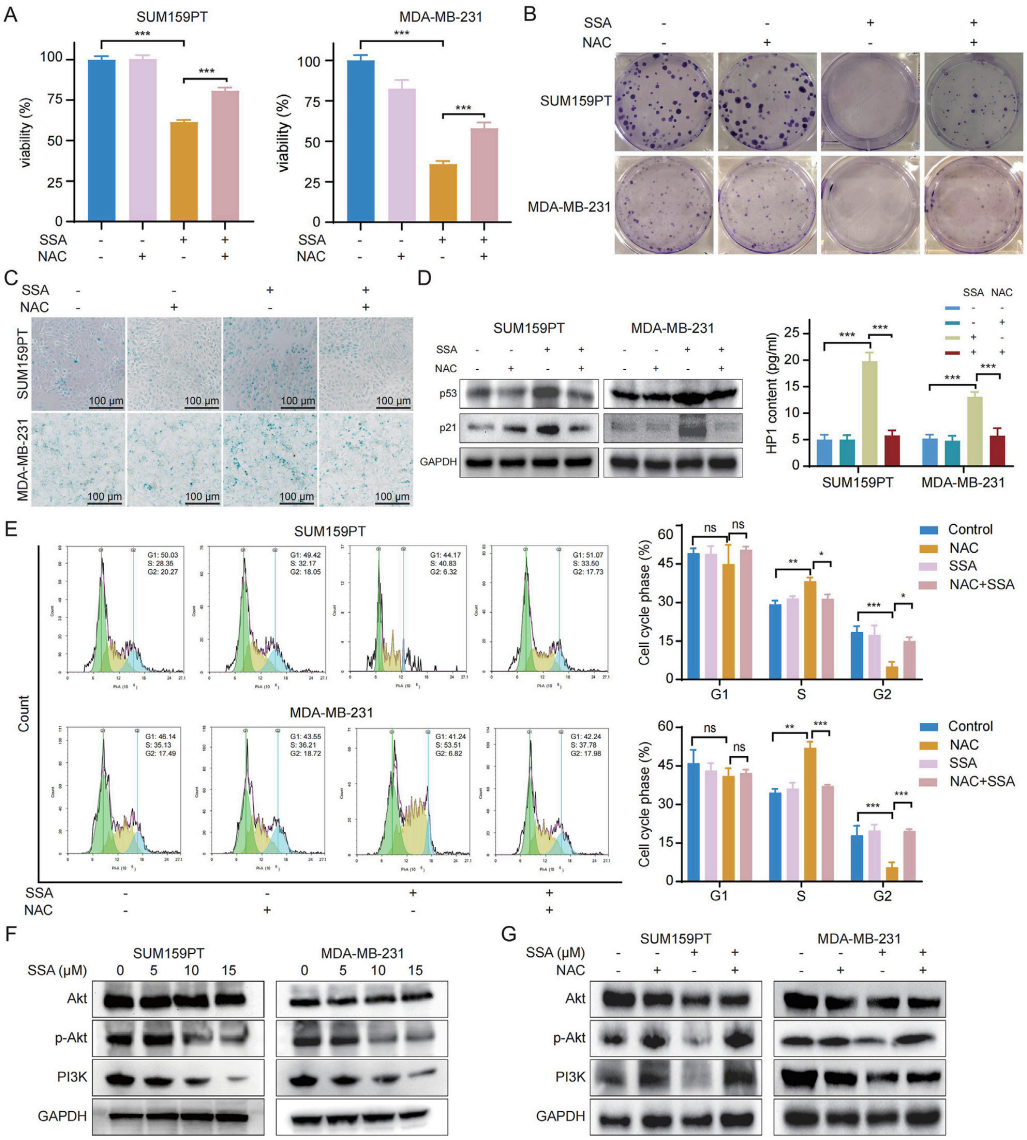
Activated ROS generation mediated the pharmacological activities of SSA in TNBC. SUM159PT and MDA-MB-231 cells were treated with NAC (2 mM) in the presence or absence of 10 μM SSA for 48 h (n = 3). Cell growth and tumorigenesis was determined by Cell Counting Kit 8 assay **(A)** and colony formation **(B)**. **(C)** Cellular senescence was detected by SA-β-gal staining. **(D)** Immunoblot analysis and ELISA of p53, p21, and HP1 expressions in SUM159PT and MDA-MB-231 cells (n = 3). **(E)** Cell cycle was analyzed by PI staining in the absence or presence of the indicated concentrations of SSA and NAC for 48 h (n = 3). **(F,G)** Immunoblot analysis of Akt, p-Akt, and PI3K expressions in SUM159PT and MDA-MB-231 cells (n = 3). All the data are presented as the means ± SD. ^*^ *P* < 0.05, ^**^ *P* < 0.01, ^***^ *P* < 0.001.

To investigate the crucial role of ROS in the pharmacological activities of SSA, N-acetylcysteine (NAC), the ROS scavenger, was applied to TNBC cells. As shown in Figure 6A, NAC counteracted SSA-induced cell proliferation inhibition, and colony formation assay detected a similar increase in cell proliferation of SSA-treated TNBC cells coupled with NAC (Figure 6B; Supplementary Figure S1C). Furthermore, we examined whether ROS generation is involved in SSA-regulated cellular senescence. As illustrated in Figure 6C and Supplementary Figure S1D, the pronounced induction of cellular senescence by SSA was significantly attenuated in the presence of NAC, as evidenced by decreased protein expressions of p53 p21, and HP1 (Figure 6D). This finding was also confirmed by increased subG2-phase and decreased subS-phase of TNBC cells under combined treatments of SSA and NAC (Figure 6E).

The PI3K/Akt pathway is recognized as a pivotal ROS-regulated pathway, exerting a significant role in the advancement of cancer through the stimulation of cancer cell proliferation (Wen et al., 2019). Additionally, the potential mechanisms of SSA in the context of TNBC, revealed by KEGG pathway analysis in Figure 2E, are ascribed to the modulation of the PI3K/Akt signaling pathway. Recently, ROS has been reported to repress the PI3K/Akt pathway (Cao et al., 2009). Thus, we detected the phosphorylated PI3K and its downstream gene Akt and p-Akt through Western blot analysis. In SSA-treated TNBC cells, a marked reduction in the protein expressions of phosphorylated PI3K and Akt was observed (Figure 6F). Moreover, NAC treatment markedly abrogated the restrained PI3K/Akt signaling by SSA (Figure 6G). Taken together, these results revealed that ROS accumulation is playing a crucial role in the pharmacological activities of SSA.

### 3.7 SSA inhibits the growth of TNBC cell xenografts, triggers cellular senescence, and suppresses of PI3K/Akt signaling pathway *in vivo*

**FIGURE 7:**
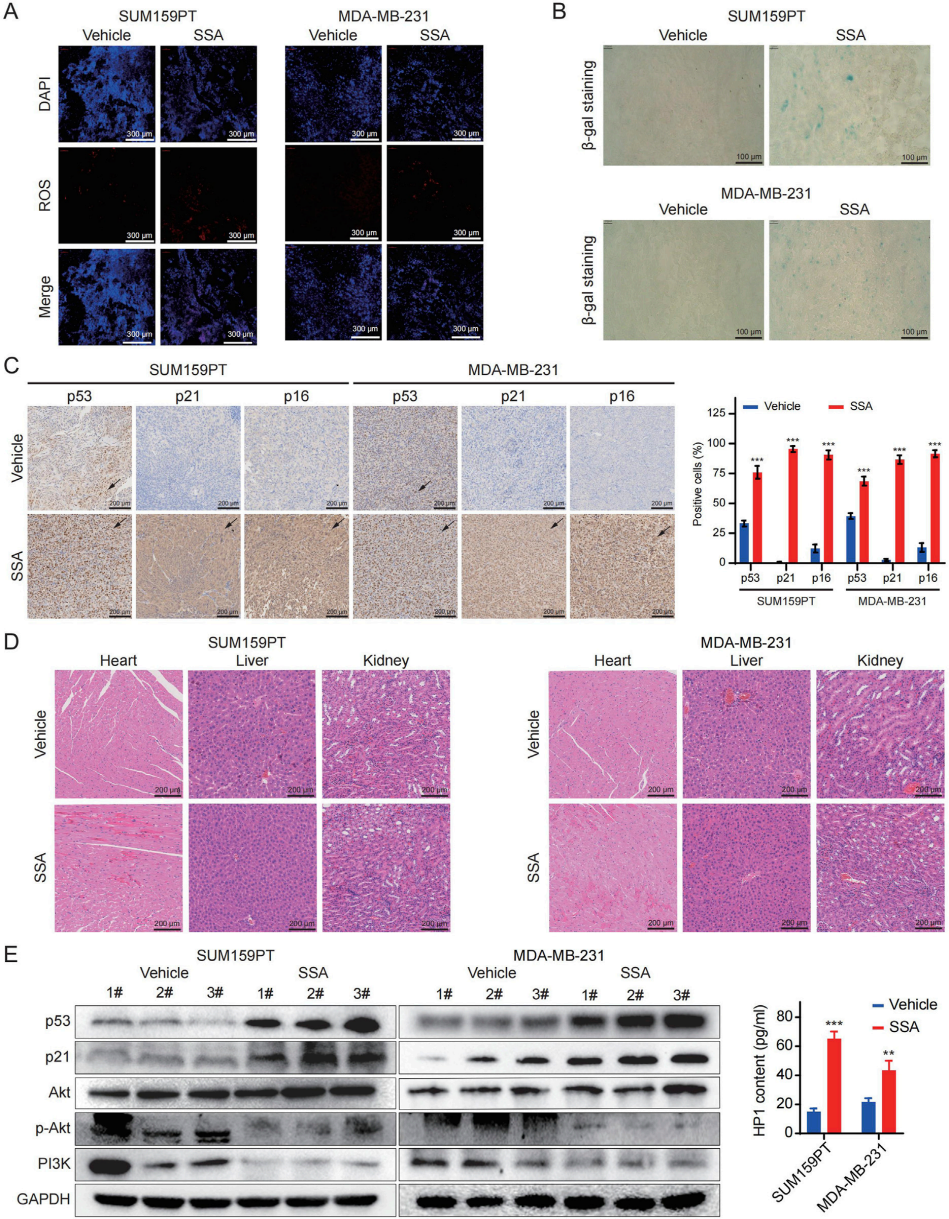
SSA inhibits the growth of TNBC cell xenografts, triggers cellular senescence, and suppresses of PI3K/Akt signaling pathway *in vivo*. **(A,B)** The ROS Immunofluorescence and SA-β-gal staining results in the SSA-treatment and the vehicle groups were compared. **(C)** p53, p21 and p16 immunohistochemistry results in the SSA-treatment and the vehicle groups were compared (n = 3). **(D)** Hematoxylin and eosin (H&E) staining of the heart, liver, and kidney collected from the mice in the treatment and the vehicle groups. **(E)** Immunoblot analysis and ELISA of p53, p21, Akt, p-Akt, PI3K, and HP1 expressions in SSA-treatment and the vehicle groups (n = 3). All the data are presented as the means ± SD. ^*^ *P* < 0.05, ^**^ *P* < 0.01, ^***^ *P* < 0.001.

Drawing from the observations in SSA-treated TNBC cells, we proceeded to evaluate the alteration of cellular senescence and the PI3K/Akt signaling pathway *in vivo* under SSA treatment. As illustrated in Figure 7A, mice subjected to SSA treatment exhibited an activated cellular senescence phenotype compared to those receiving the vehicle control via β-gal staining. Subsequent evaluation of senescence indices through p21 and p53 immunohistochemical analyses and HP1 ELISA (Figures 7B,E). Western blot analyses further supported that SSA effectively inhibits the PI3K/Akt signaling pathway (Figures 7C,E). This finding underscores the pivotal role of the PI3K/Akt pathway in mediating the response of TNBC to SSA-induced senescence and modulation of proliferation. Importantly, SSA administration demonstrated no discernible toxic effects on the animals, as evidenced by the unaltered pathological morphology of major organs (Figure 7D). Taken together, these data validate the therapeutic efficacy and safety profile of SSA as an anticancer agent.

## 4 Discussion

The pervasive incidence of diverse cancer-inducing genetic mutations leads to the inadequacy of current cancer treatment modalities, thus intensifying the global scientific pursuit for anticancer agents capable of modulating multiple signaling pathways. Natural products, esteemed for their rich legacy in offering essential molecular frameworks for drug discovery, have attracted heightened focus for their potential in cancer treatment (Huang et al., 2021). To date, several widely applied anticancer drugs in clinics are sourced from natural products, including irinotecan, vincristine, etoposide, paclitaxel, et al. Saikosaponin A, extracted from Radix Bupleuri, has demonstrated potent anti-TNBC properties, as evidenced by MTT, EdU, and colony formation assays. Further investigations revealed that ROS level, cellular senescence, and cell cycle arrest were induced in response to SSA treatment. Additionally, SSA was found to inhibit the PI3K/Akt signaling pathway in TNBC cells. These findings are further corroborated by *in vivo* study. Previous studies have never reported that SSA can induce senescence in breast cancer cells, and this biological activity of SSA has been discovered for the first time in our study.

A growing number of studies have underscored the anticancer properties of active metabolites isolated from Radix Bupleur. Among nearly 100 different triterpenoid saponins purified from Radix Bupleuri, Saikosaponins are considered as key contributors to the comprehensive pharmacological activities of Radix Bupleur. Given the similar basal chemical structures, the Saikosaponins collectively exhibit regulatory roles across a spectrum of pharmacological activities, mechanisms, models, and applications. SSB2 was reported to exhibit antitumor activity in several cancer cell lines, especially against melanoma (Kim, 2018). Hsu et al. (2004) reported that Saikosaponin D could decrease cell proliferation and induce apoptosis in hepatoma cells via suppression of the signal transducer and activator of transcription 3- (STAT3-) hypoxia-inducible factor- (HIF-) 1α-Cox-2 pathway. Differing from other Saikosaponins, SSC exhibited therapeutic potential targeting endothelial cells instead of cancer cells (Shyu et al., 2004). As a major form of Saikosaponins, the anti-cancer potential of SSA has been extensively reported (Kang et al., 2017). It is revealed by Sheng (Wen-Sheng, 2003) that SSA could inhibit hepatoma growth via induction of Erk and its downstream p16 family proteins. Additionally, SSA was reported to trigger apoptosis of breast cancer cells in previous studies, although the detailed mechanisms underlying this effect remain to be fully elucidated (Chen et al., 2003). In this study, our group has investigated not only the SSA-induced cytotoxicity in TNBC cells, but also the impacts of SSA on cell cycle, cellular senescence, and ROS production. Furthermore, the PI3K/Akt signaling pathway was significantly inhibited in response to SSA treatment. Given the vast range of pharmacological activities of SSA, our research holds considerable significance in elucidating the anti-cancer potential of SSA, promoting the development of SSA-based therapeutic strategies.

Cellular senescence represents a stress-induced condition marked by terminal cell cycle arrest, diverse macromolecular alterations, and a hypersecretory, pro-inflammatory phenotype (Schmitt et al., 2022). Senescent cells have been revealed by Campisi and Hornsby’s group for their involvement in the malignant conversion of otherwise non-malignant cells, as well as the proliferation of fully transformed breast cancer cells (Liu and Hornsby, 2007). Similarly, drug-activated systemic senescence has been shown to promote breast cancer metastasis *in vivo* (Demaria et al., 2017). Intriguingly, in other studies, senescence has emerged as an anti-tumor effector mechanism. Indeed, as a stable state of cell cycle arrest, senescent cells function as a natural barrier to tumorigenesis. Additionally, accumulated studies have addressed the significance of senescent cells in triggering cancer immunosurveillance. As an activated signal in senescence, p53 restoration triggered innate immune response via the SASP factors colony stimulating factor 1 (CSF1), C-C motif chemokine ligand 2 (CCL2), Interleukin-15 (IL-15), and C-X-C motif chemokine ligand 1 (CXCL1), thereby contributing to tumor clearance (Xue et al., 2007). In another study, p21-dependent senescence demonstrated anti-tumor activity mediated by chemokine (C-X-C motif) ligand 14 (CXCL14) and insulin-like growth factor binding protein 3 (IGFBP3) (Sturmlechner et al., 2021). Our work primarily confirmed the dose-dependent cytotoxicity of SSA in TNBC cells, and further exploration revealed that SSA could significantly trigger cellular senescence evidenced by positive SA-β-Gal staining, SASP induction, and upregulation of p53 and p21. This suggests that a tumor-suppressive function of senescence is activated in response to SSA treatment (Perillo et al., 2020).

**FIGURE 8:**
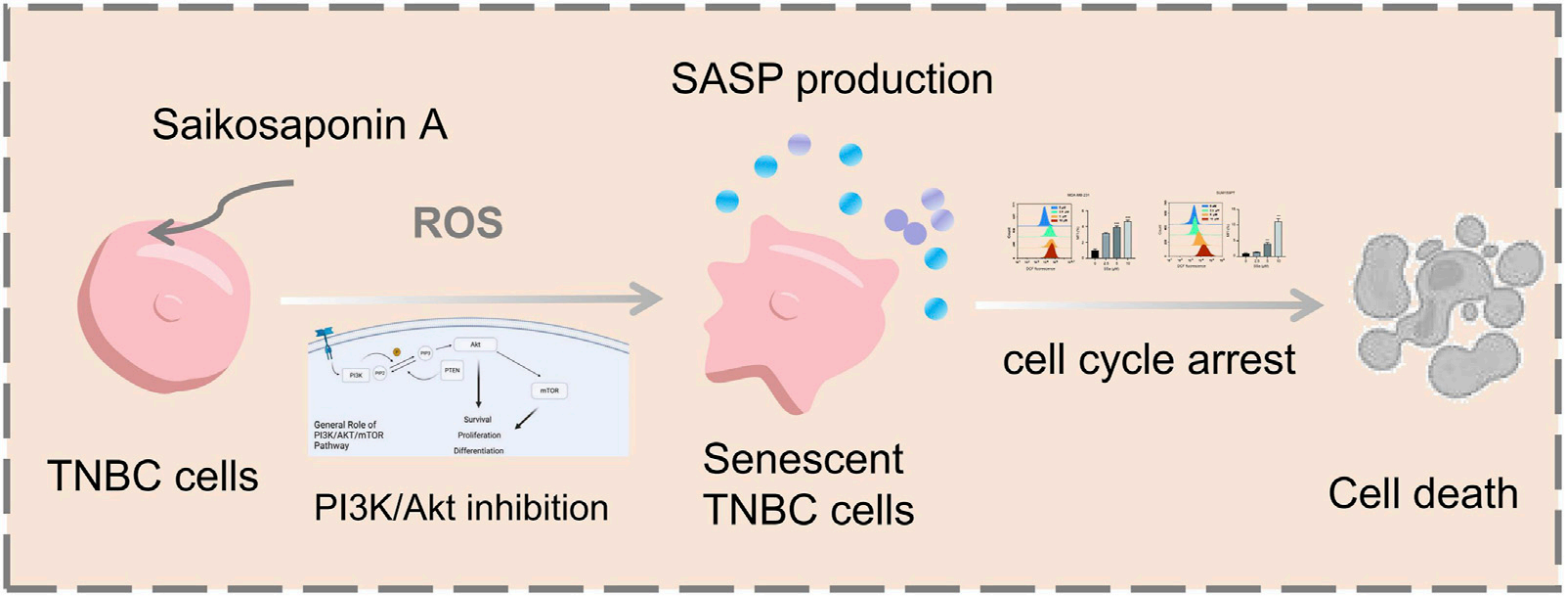
Schematic illustration of the potential underlying mechanism responsible for SSA-induced cellular senescence.

Numerous anticancer drugs exert their therapeutic effects via oxidative stress activation, resulting in accumulated intracellular ROS. In this study, we revealed that SSA could trigger cellular ROS production in TNBC cell lines. More importantly, ROS are regarded as second messengers in regulating the PI3K/Akt signaling pathway, which is involved in many cellular activities, such as cell senescence, cell apoptosis, and cell proliferation (Sheppard et al., 2022). In TM3 Leydig cells, abamectin-induced ROS restoration inhibited the PI3K/Akt/mTOR cascade, leading to cell programmed death (Zhu et al., 2020). In addition, ROS have been implicated in osteoblast apoptosis and chemo-drug resistance via the PI3K/Akt signaling pathway (Zhang et al., 2020). Herein, both *in vivo* and *in vitro* studies showed that the activity of PI3K/Akt pathway was significantly decreased by SSA. We speculated that ROS buildup resulted in SSA-induced cell cycle arrest and senescence of TNBC cells, as well as the restrained PI3K/Akt signaling pathway. Recent studies have also reported that SSA can target the PI3K/Akt pathway to exert anti-pancreatic and gastric cancer, and cholangiocarcinoma activity (Shi et al., 2023; Wang et al., 2023; Song et al., 2024), and it has also been reported that SSA can target the PI3K/Akt pathway for the treatment of skeletal muscle atrophy (Huang et al., 2023), but no studies have reported that SSA targeting this pathway can exert anti-TNBC activity. Our results demonstrate that the indicated activities of SSA on TNBC is compromised by reduced ROS generation. To the best of our knowledge, the current work shows the inaugural investigation into the targets of Saikosaponin A in TNBC employing network analysis approaches. Taken together, our findings reveal that SSA markedly inhibits the proliferation of TNBC cells and fosters senescence and cell cycle arrest, primarily through the induction of ROS production and the inhibition of the PI3K/Akt signaling pathway (Figure 8). This study elucidates a novel targets underlying SSA-induced toxicity in TNBC, bolstering its potential as a promising therapeutic approach for patients with TNBC.

However, network studies must critically assess the pharmacological evidence to evaluate the potential effects of a preparation/herbal (medical) product and the limitations of the evidence. This study only verified the most critical target Akt. There are other unverified potential targets of SSA, such as STAT3 and NFKB, which have been reported to induce breast cancer cell senescence (Zhou et al., 2024), and may need to be paid attention to in the practical application of SSA in the future. In addition, many times the results of cell and animal experiments are not fully reflected in humans, which is also a concern.

## 5 Conclusion

In summary, we show that SSA could effectively suppress breast cancer growth through inhibition of PI3K/Akt pathways and activation of cellular senescence. Most importantly, there was no obvious toxicity was found in treated mice, suggesting that SSA could be a promising candidate for the treatment of breast cancer in clinic.

## Data Availability

The original contributions presented in the study are included in the article/Supplementary Material, further inquiries can be directed to the corresponding author.
