# Supplementary material for: Saikosaponin A induces cellular senescence in triple-negative breast cancer by inhibiting the PI3K/Akt signalling pathway
Source: Front Pharmacol. 2025 Apr 25;16:1532579. doi: 10.3389/fphar.2025.1532579 (PMC12062077; doi:10.3389/fphar.2025.1532579)

**Western blot original images**

Figure 3F SSA-Akt

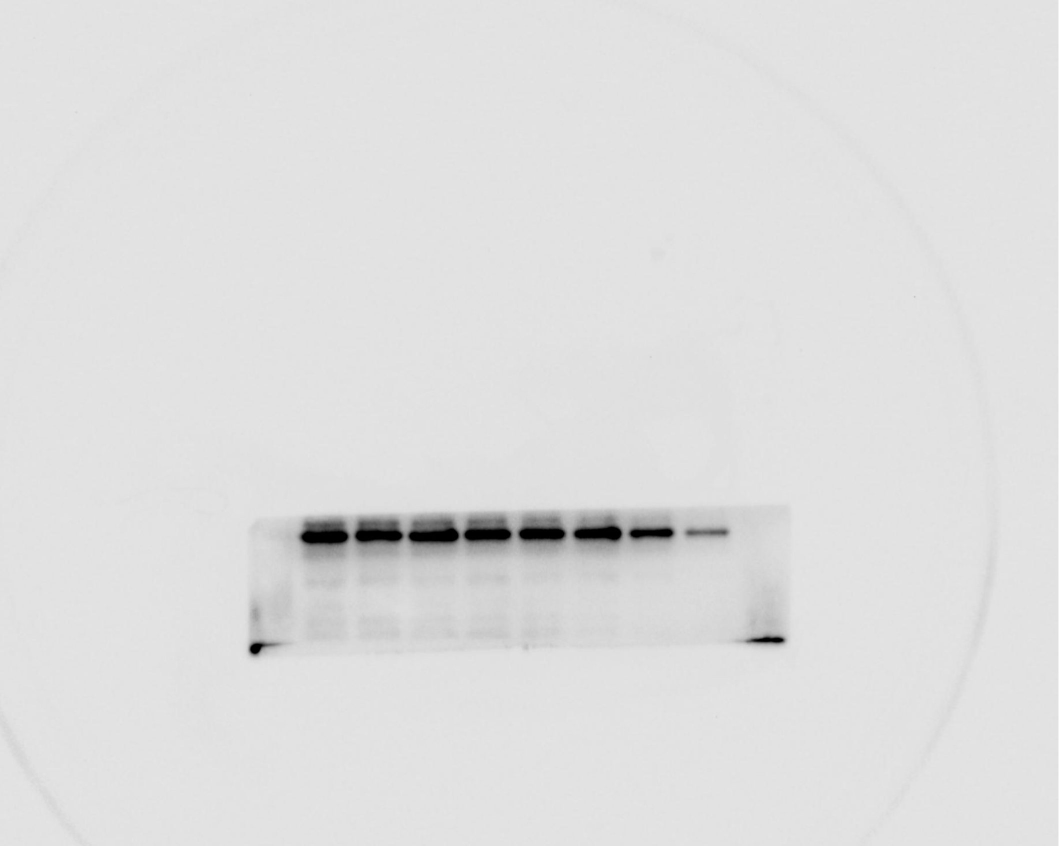

Figure 3F Blank-Akt

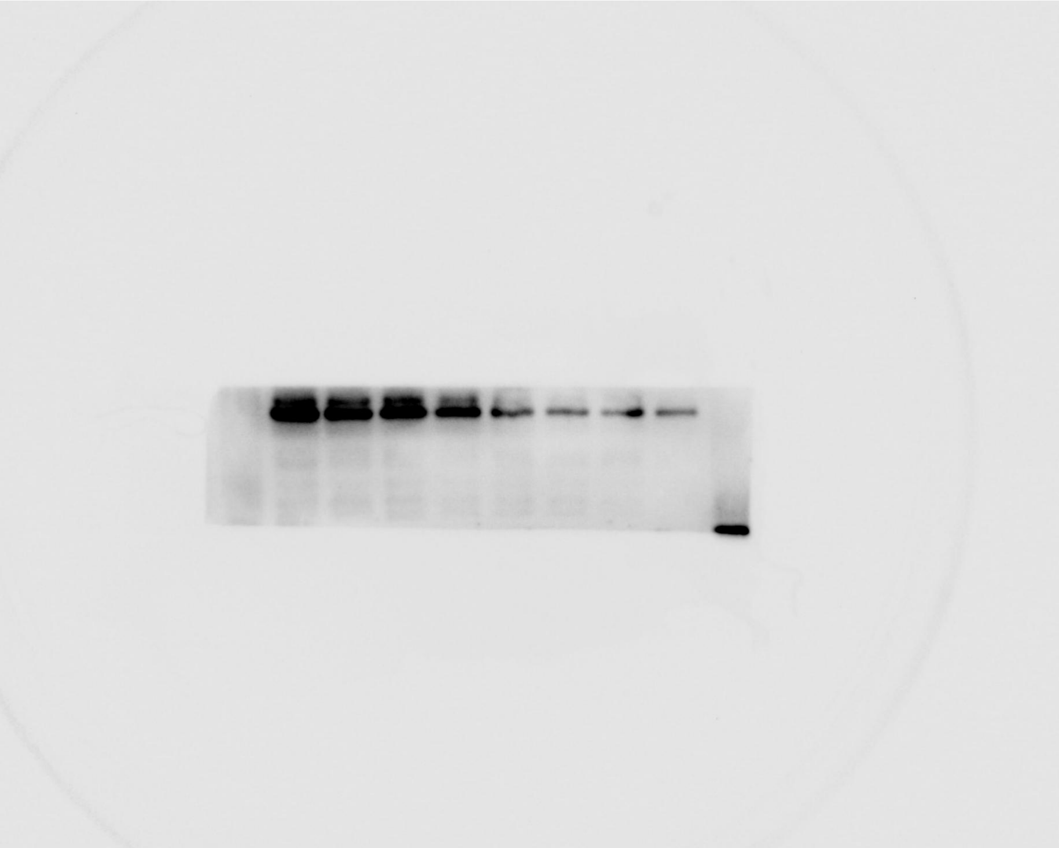

Figure 5B SUM159PT p53

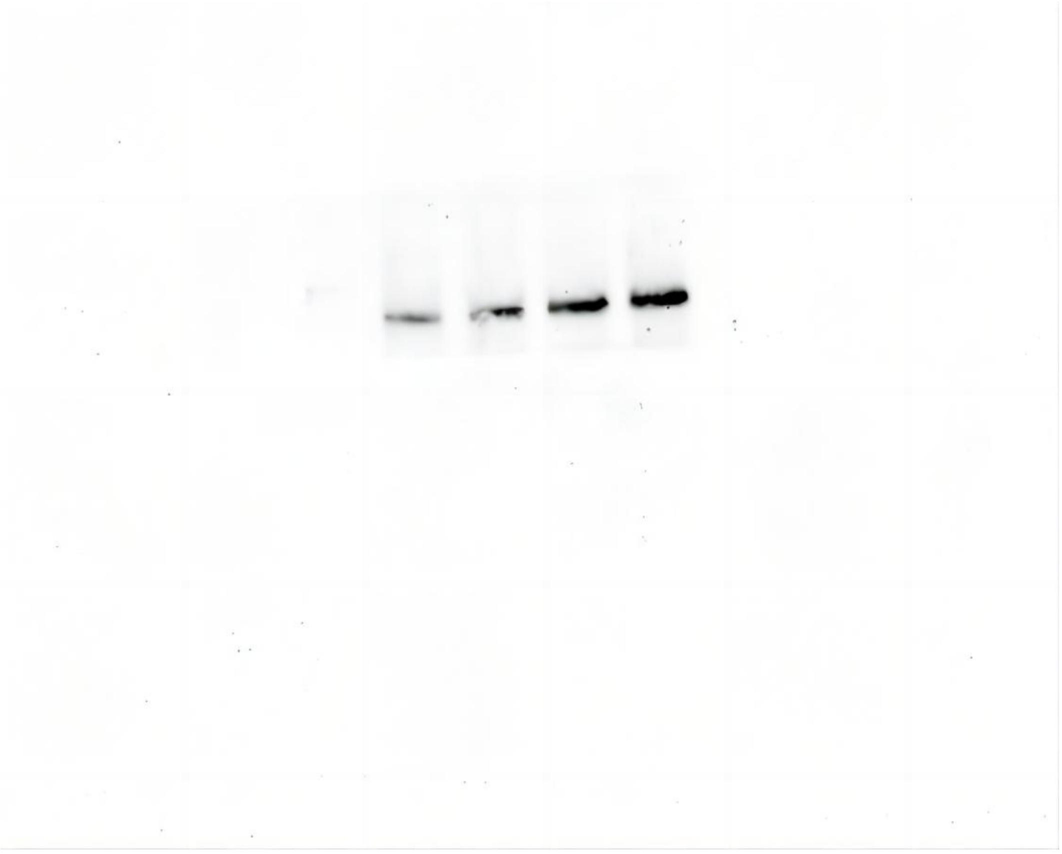

Figure 5B SUM159PT p21

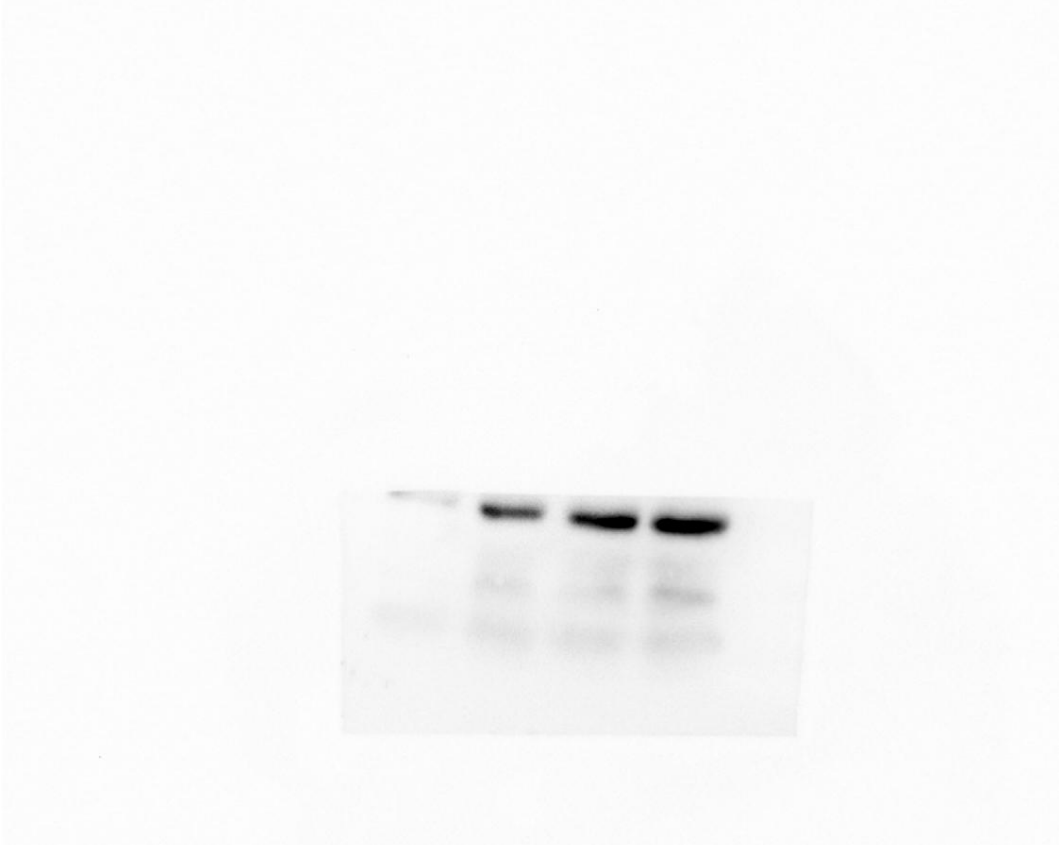

Figure 5B SUM159PT GAPDH

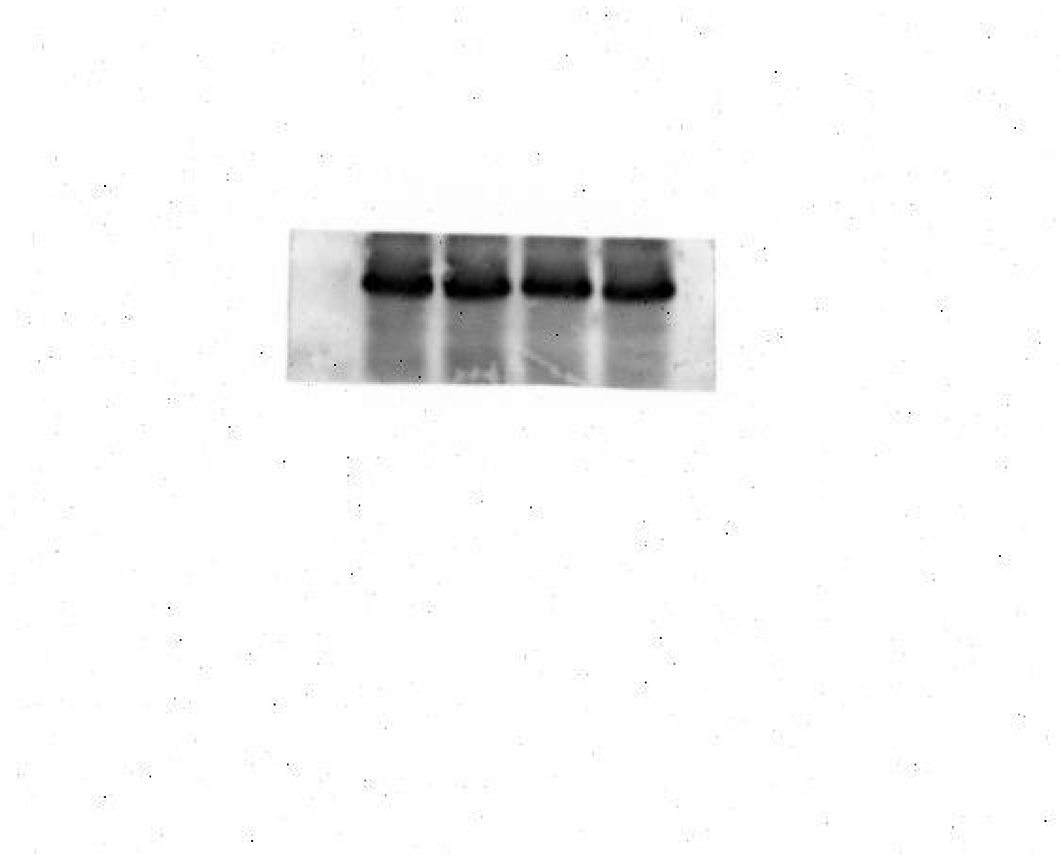

Figure 5B MDA-MB-231 p53

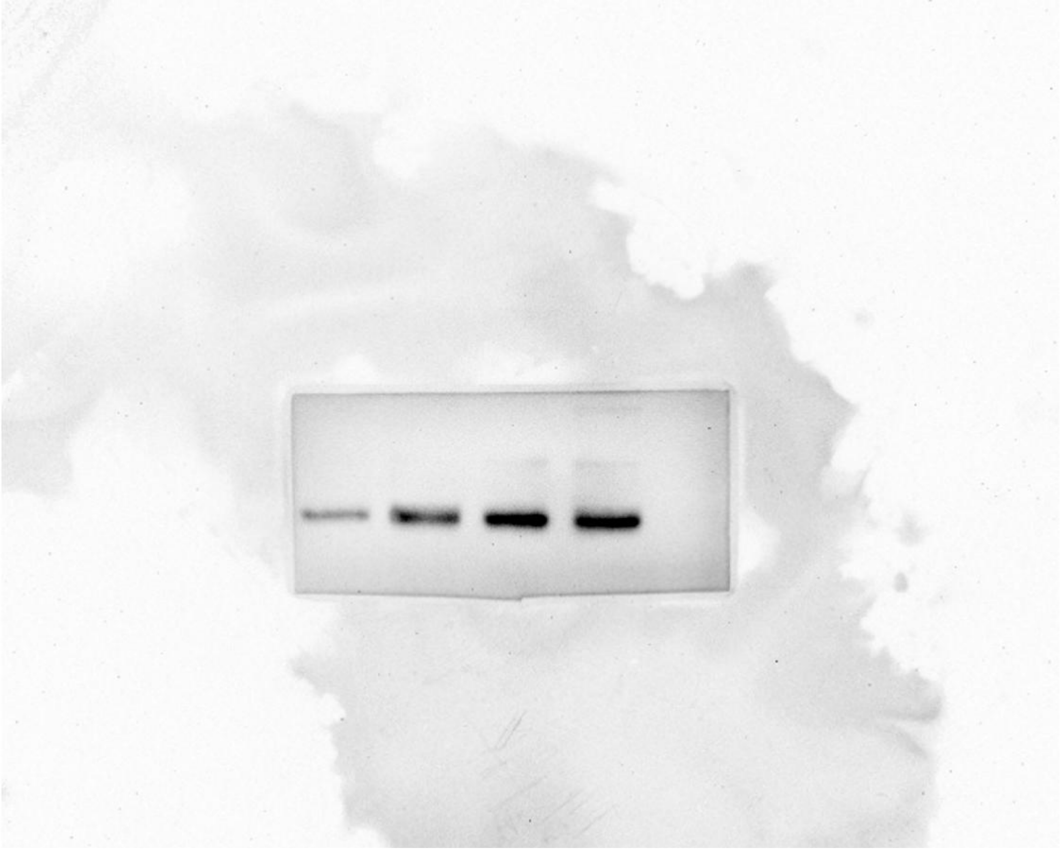

Figure 5B MDA-MB-231 p21

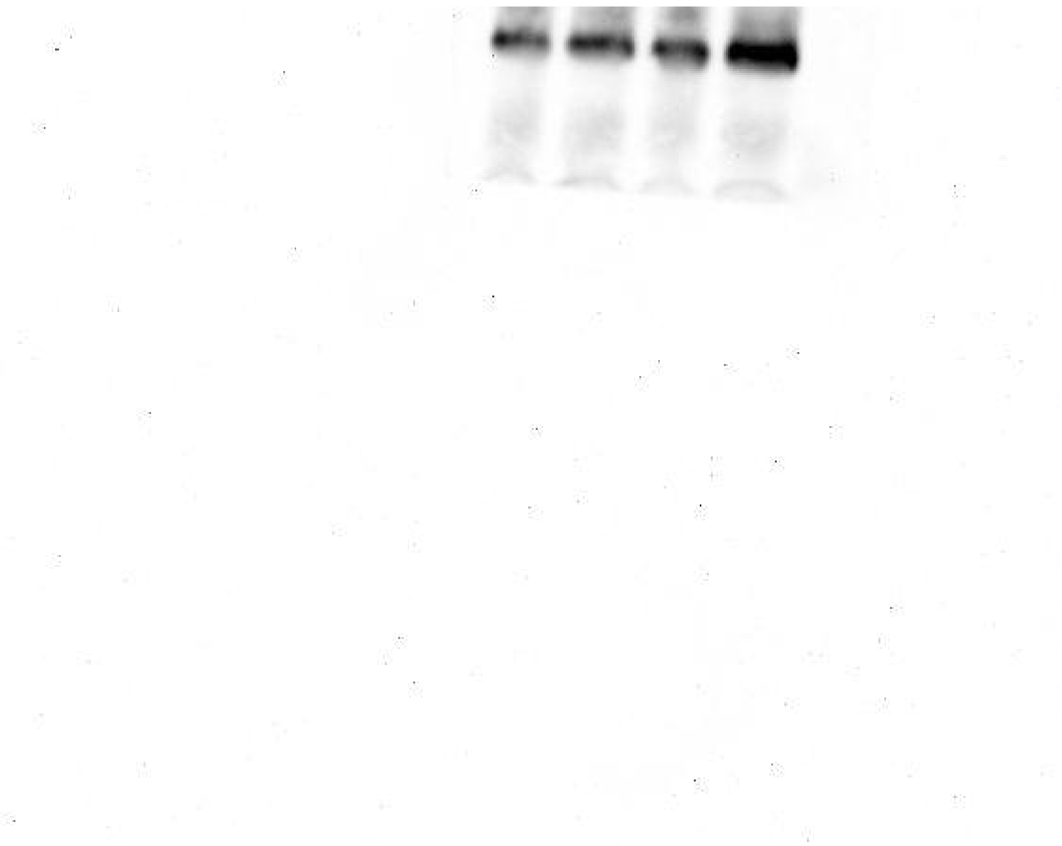

Figure 5B MDA-MB-231 GAPDH

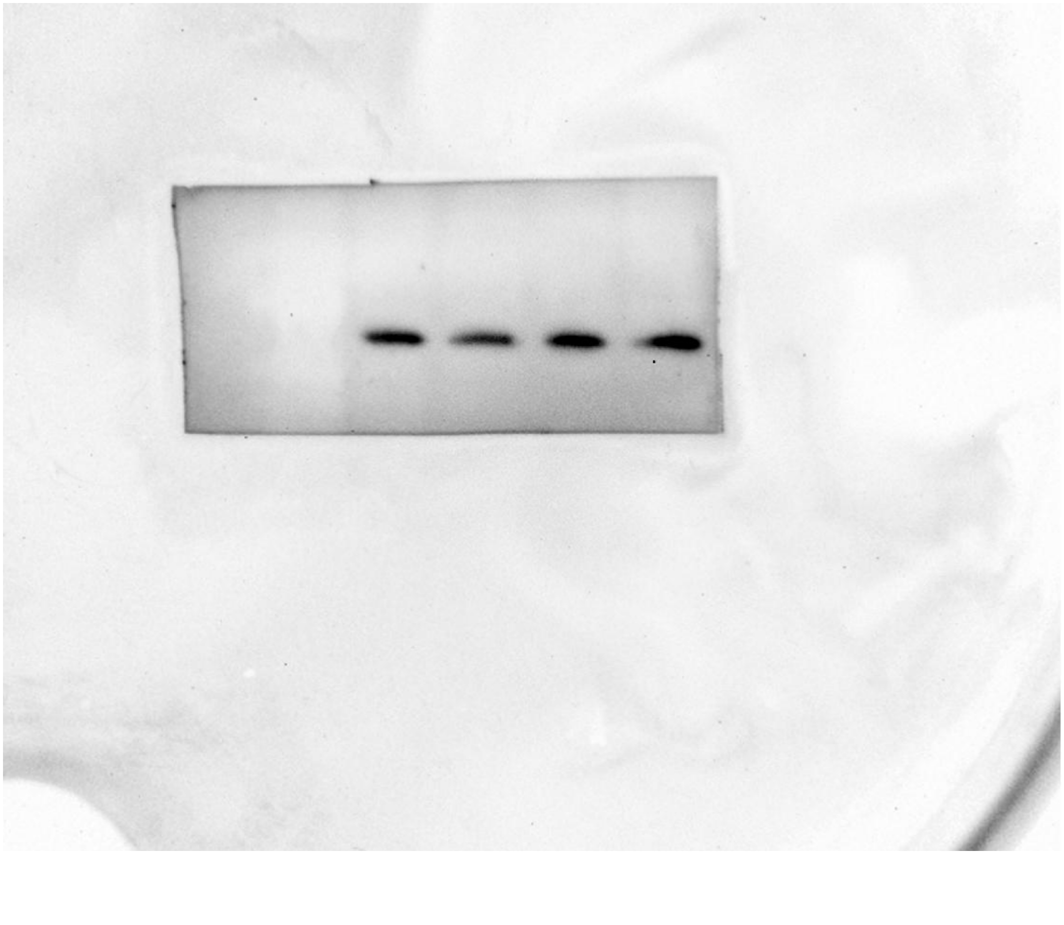

Figure 6D SUM159PT p53

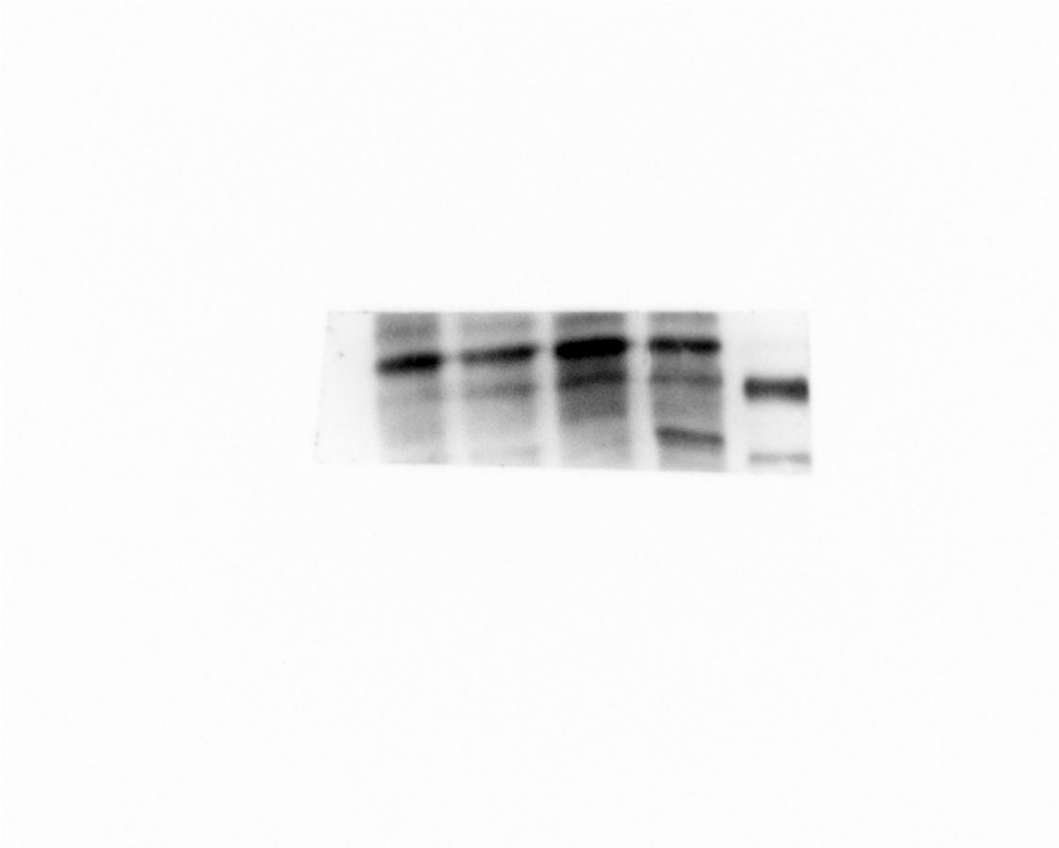

Figure 6D SUM159PT p21

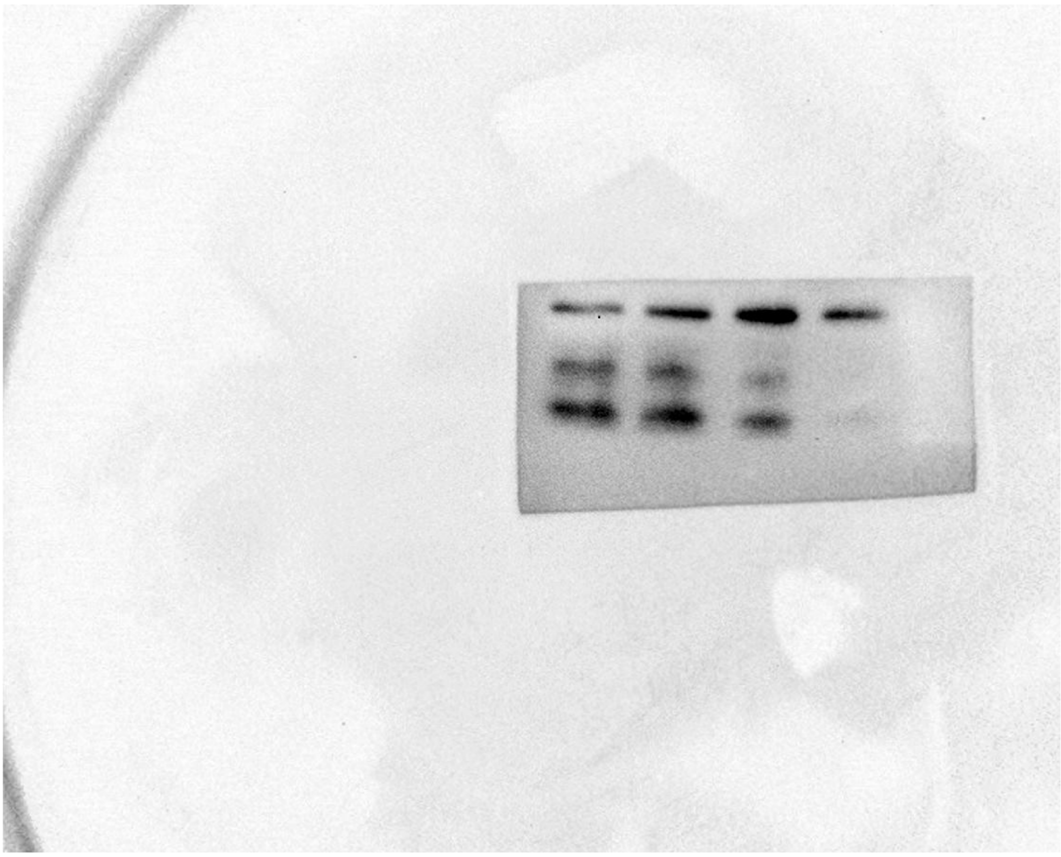

Figure 6D SUM159PT GAPDH

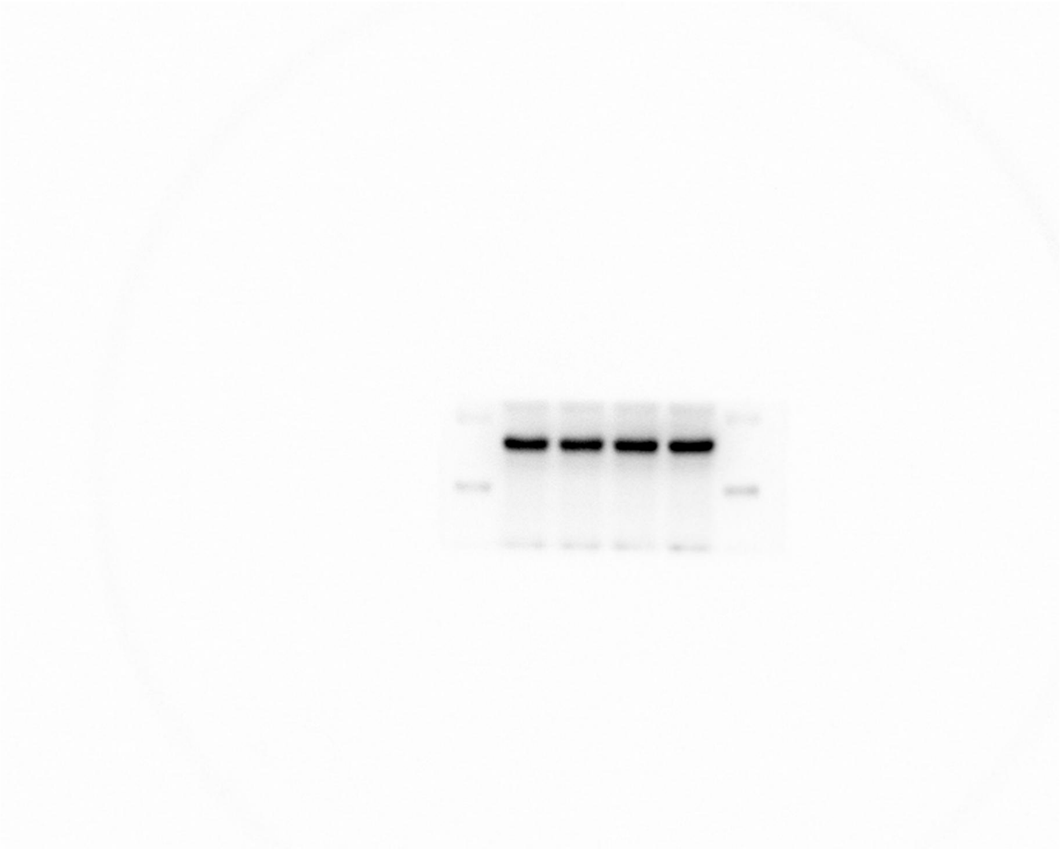

Figure 6D MDA-MB-231 p53

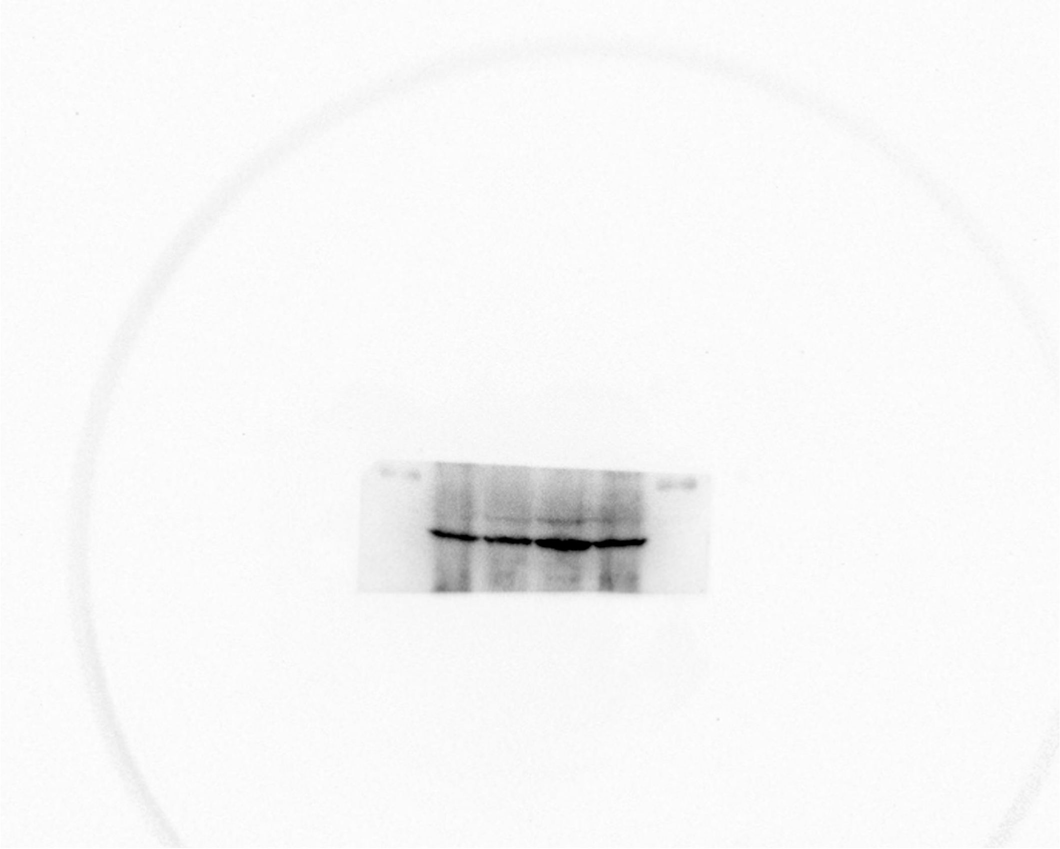

Figure 6D MDA-MB-231 p21

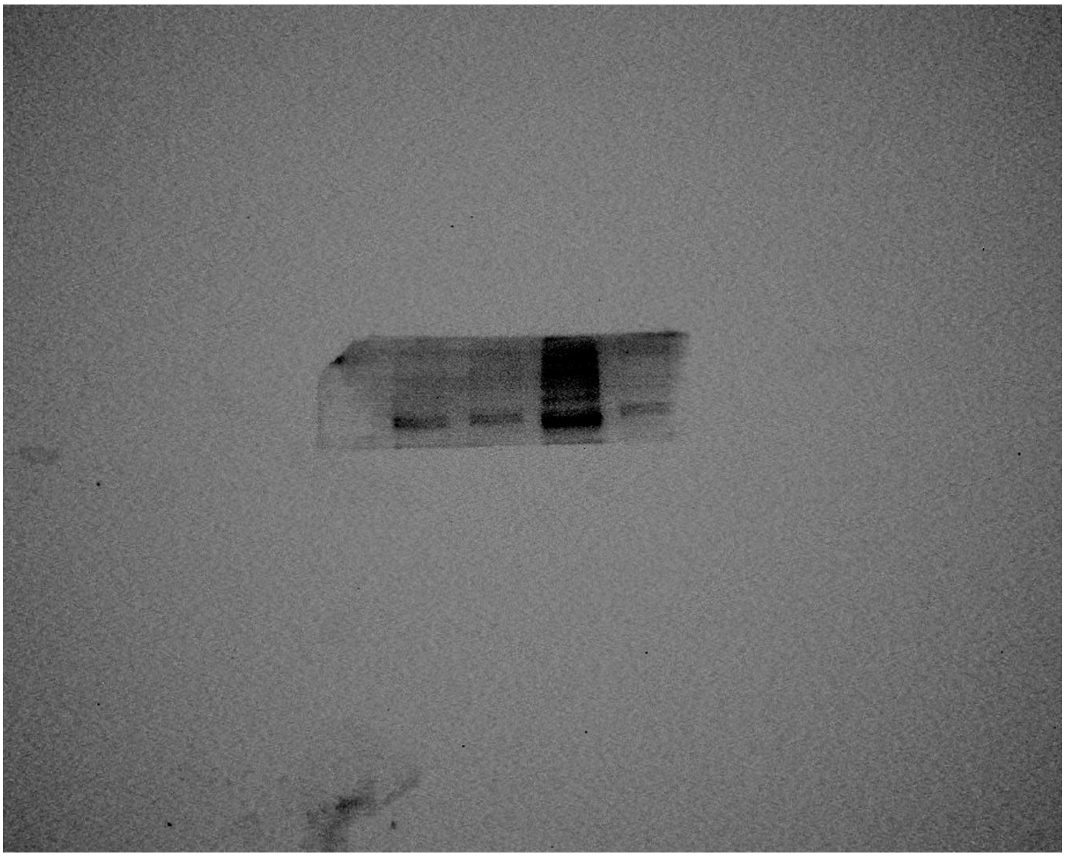

Figure 6D MDA-MB-231 GAPDH

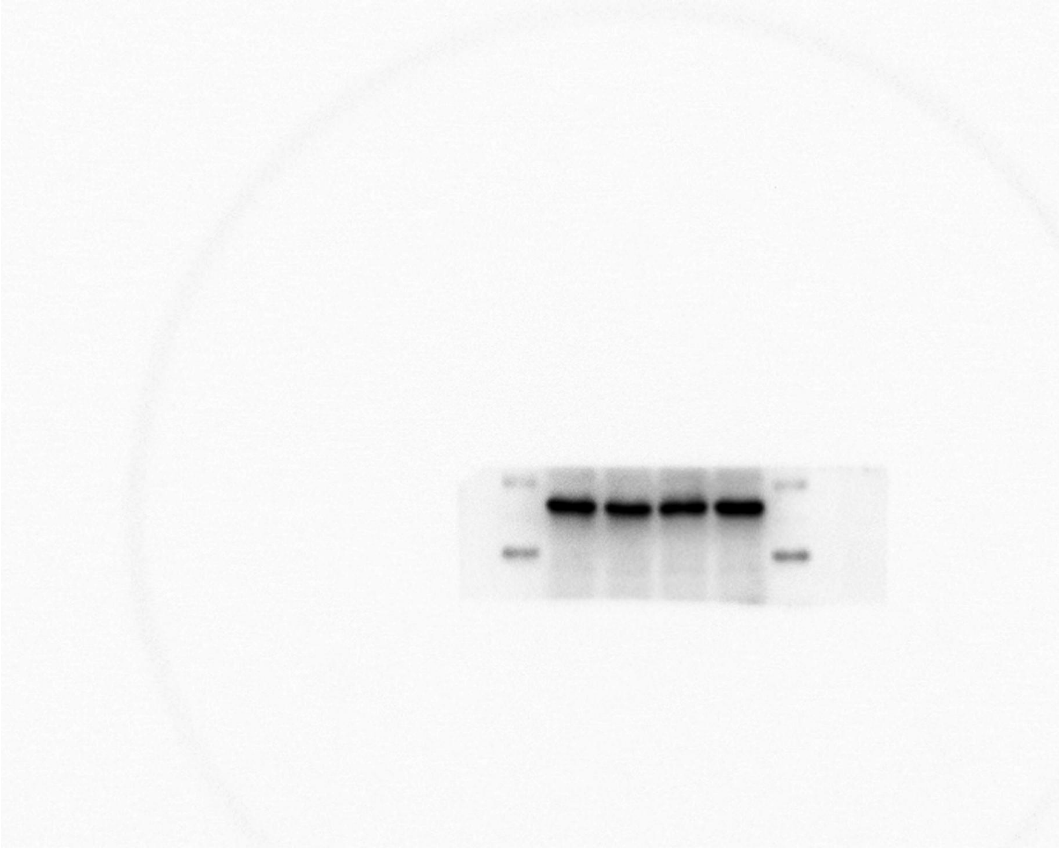

Figure 6F SUM159PT Akt

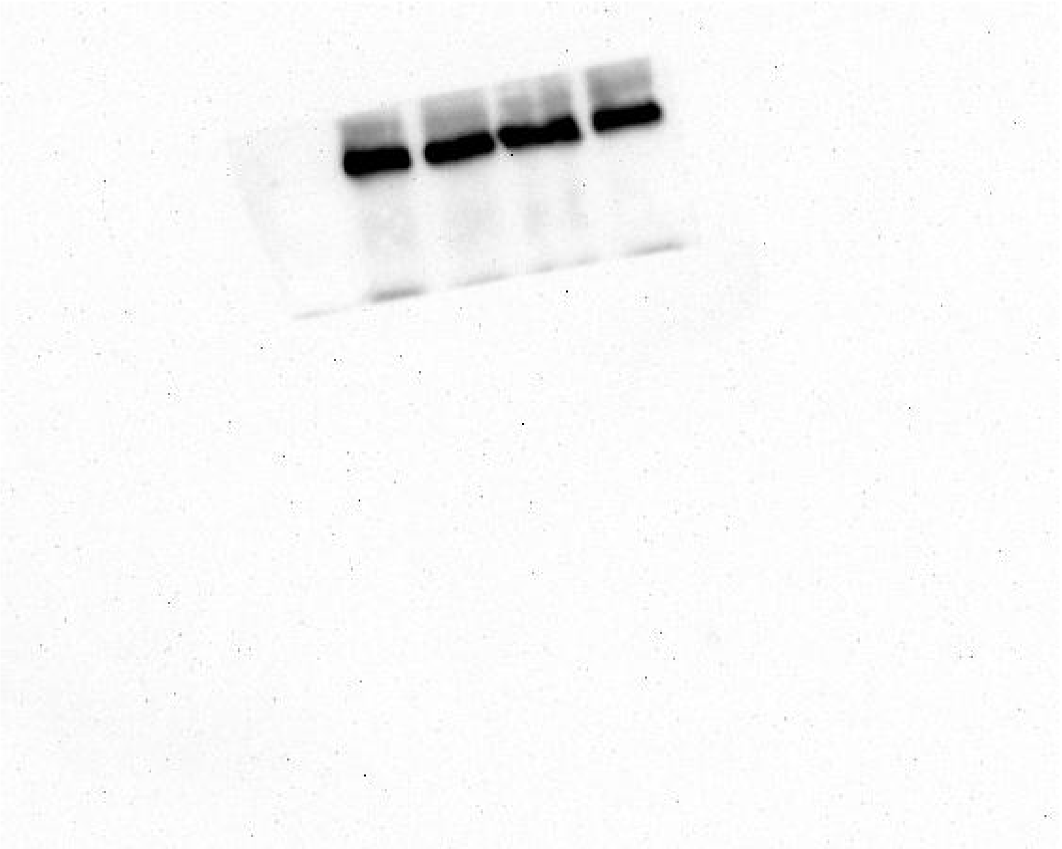

Figure 6F SUM159PT p-Akt

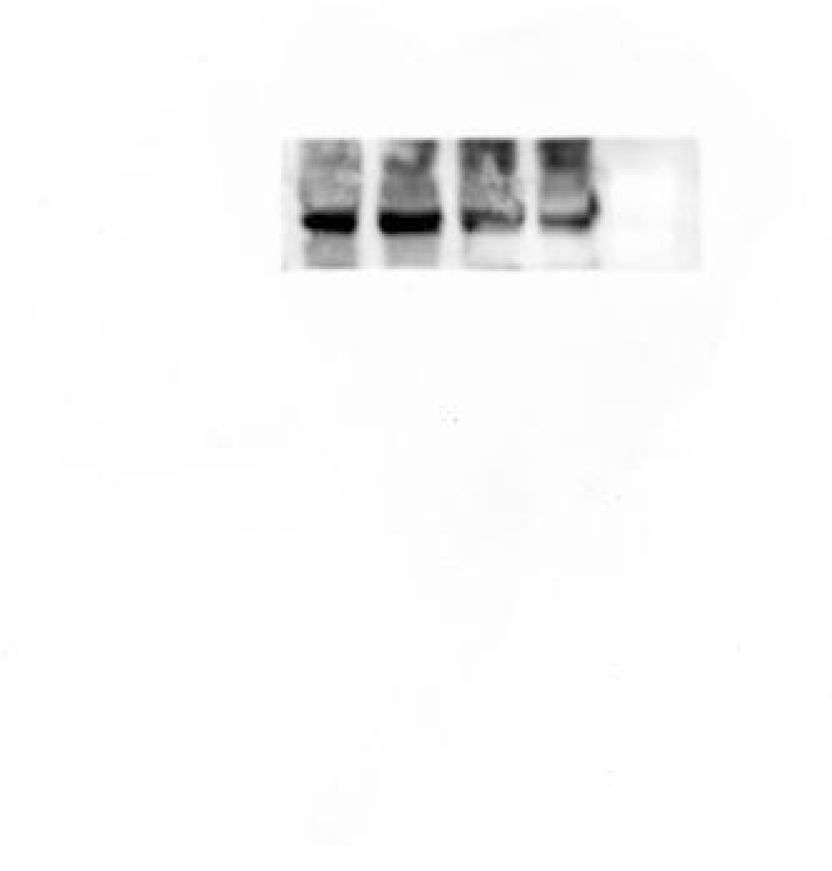

Figure 6F SUM159PT PI3K

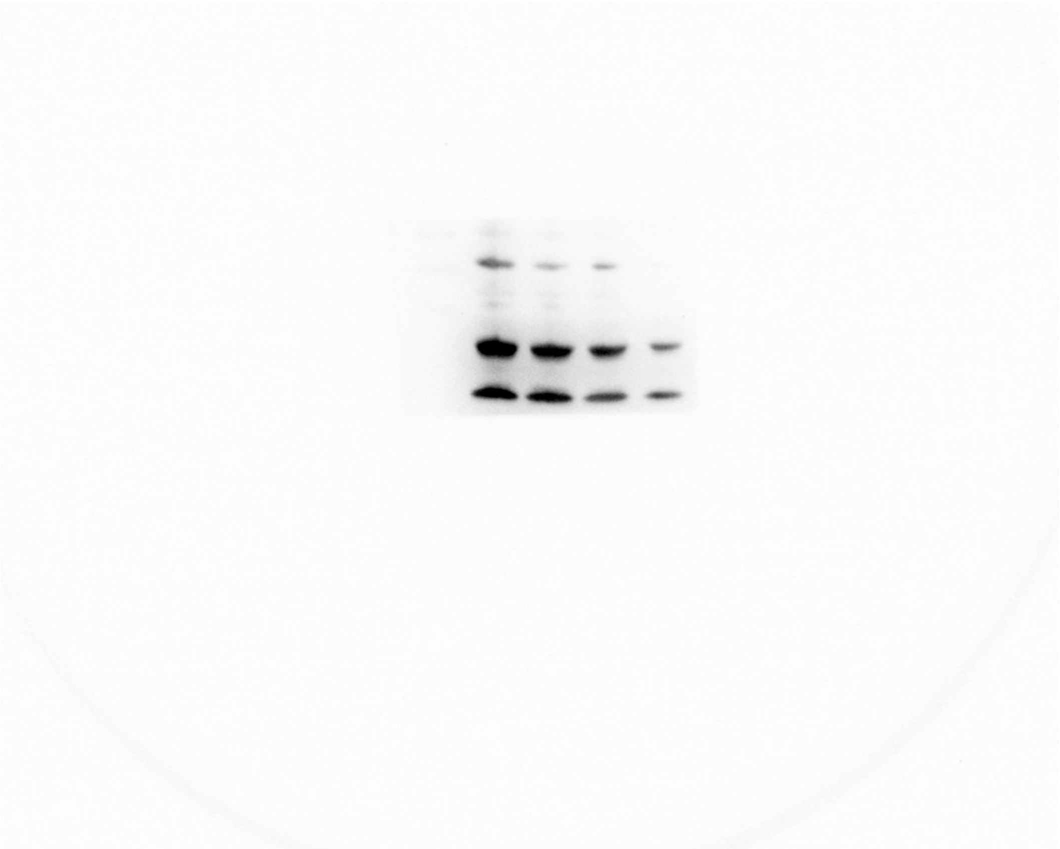

Figure 6F SUM159PT GAPDH

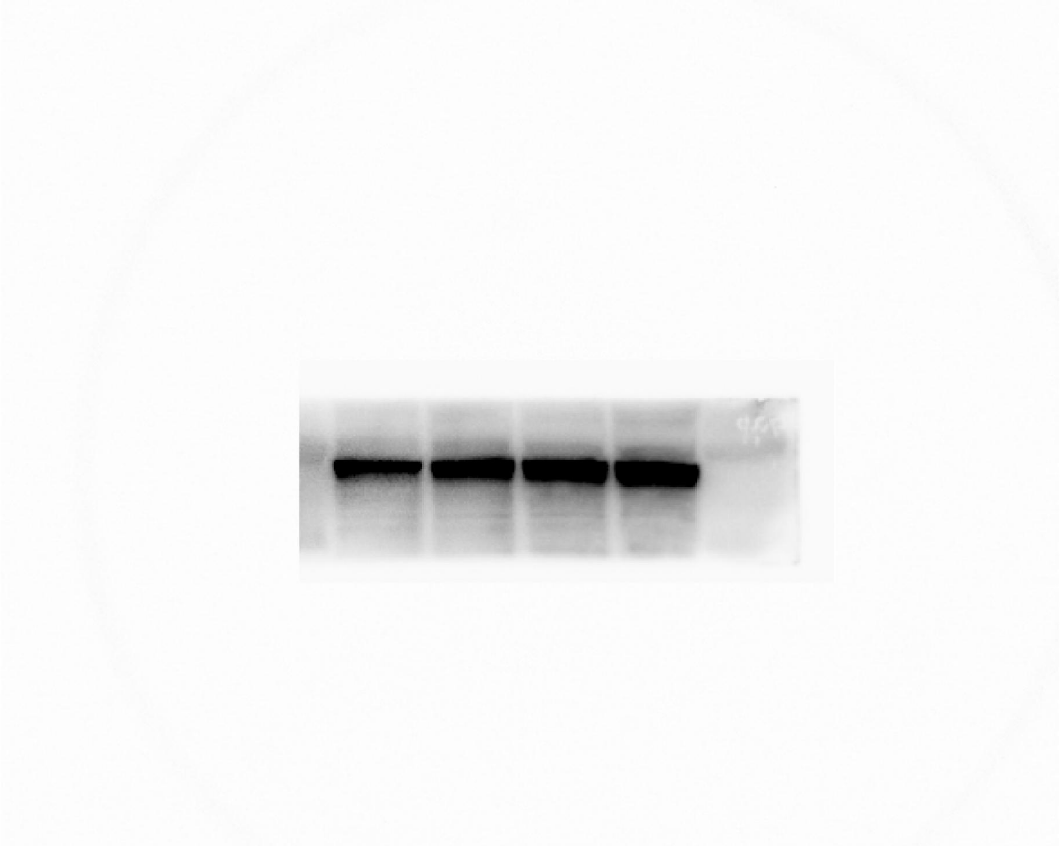

Figure 6F MDA-MB-231 Akt

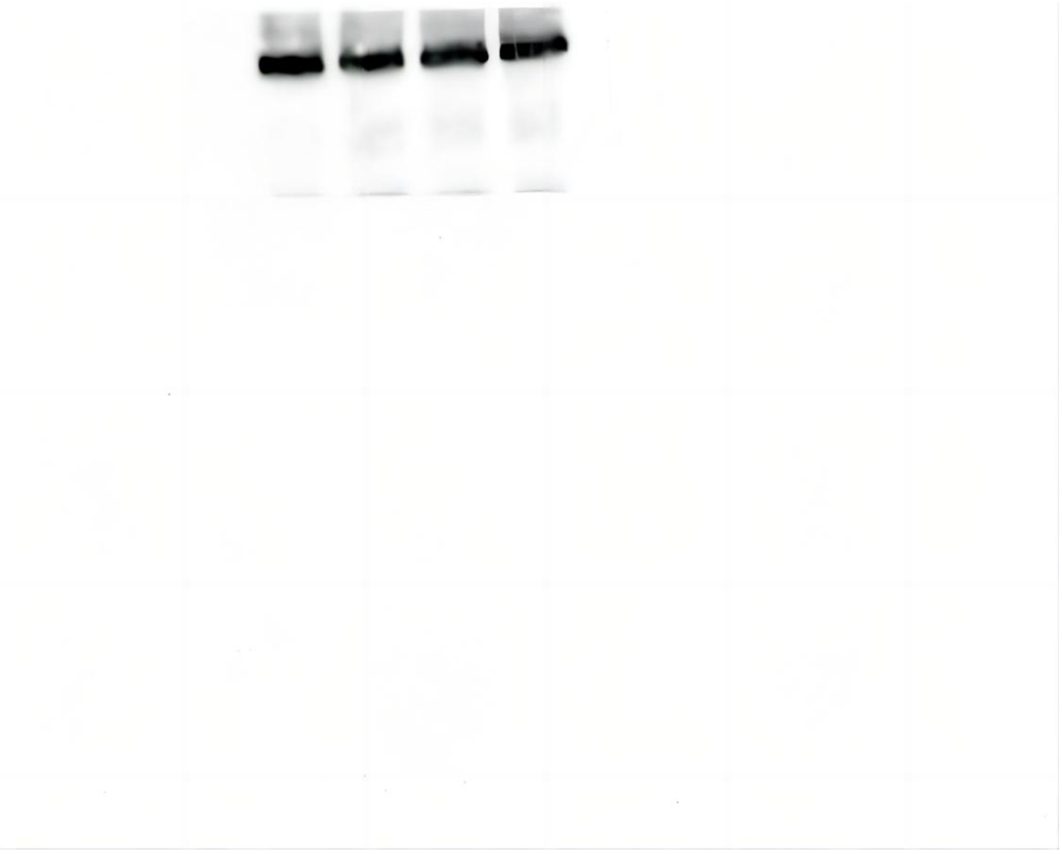

Figure 6F MDA-MB-231 p-Akt

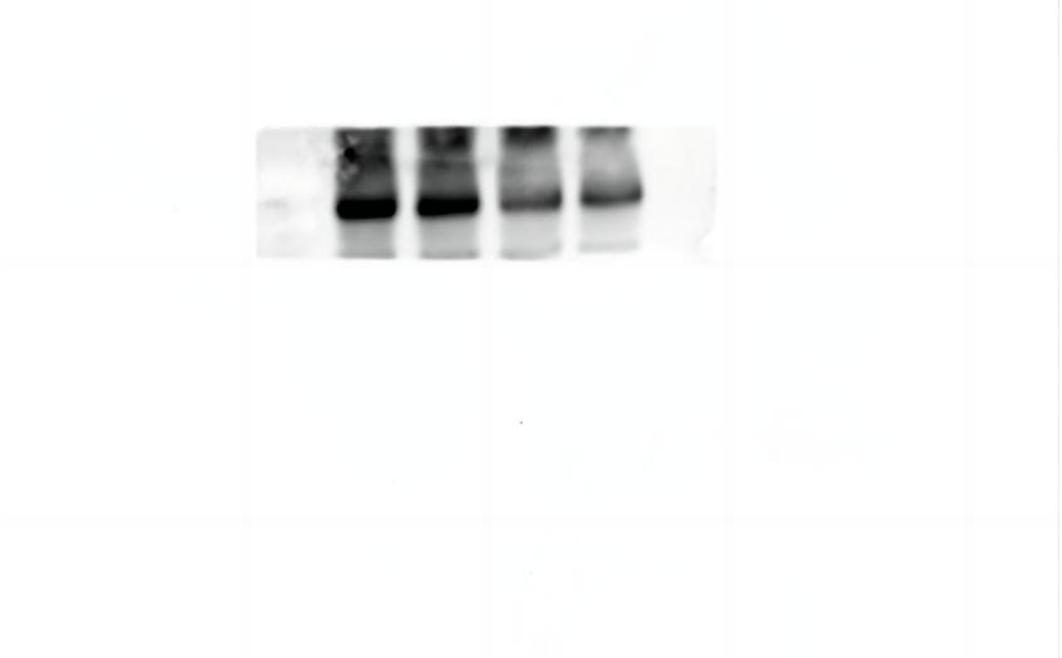

Figure 6F MDA-MB-231 PI3K

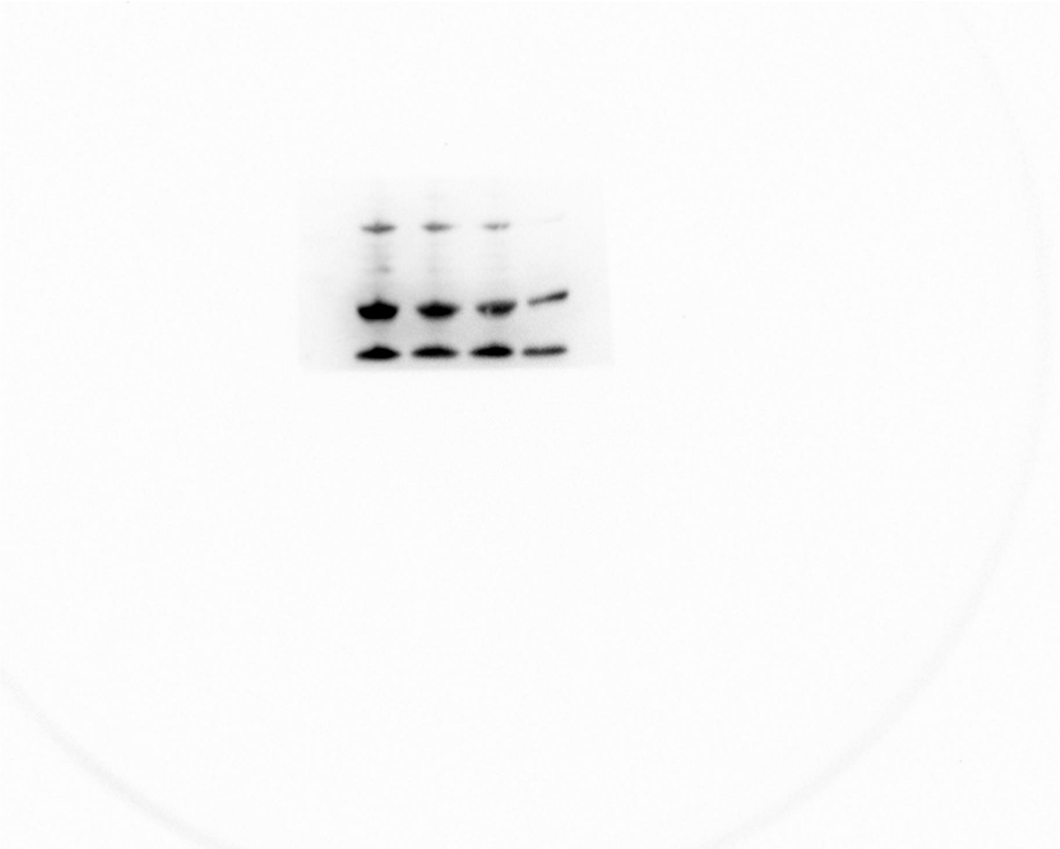

Figure 6F MDA-MB-231 GAPDH

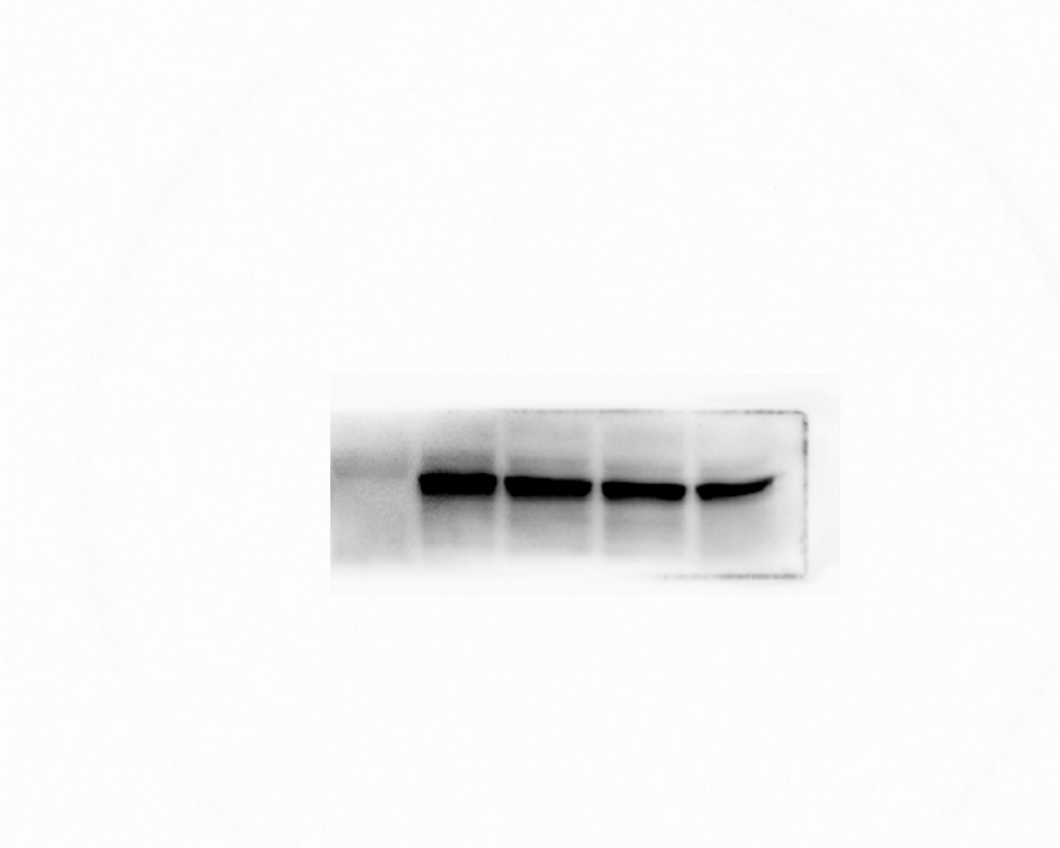

Figure 6G SUM159PT Akt

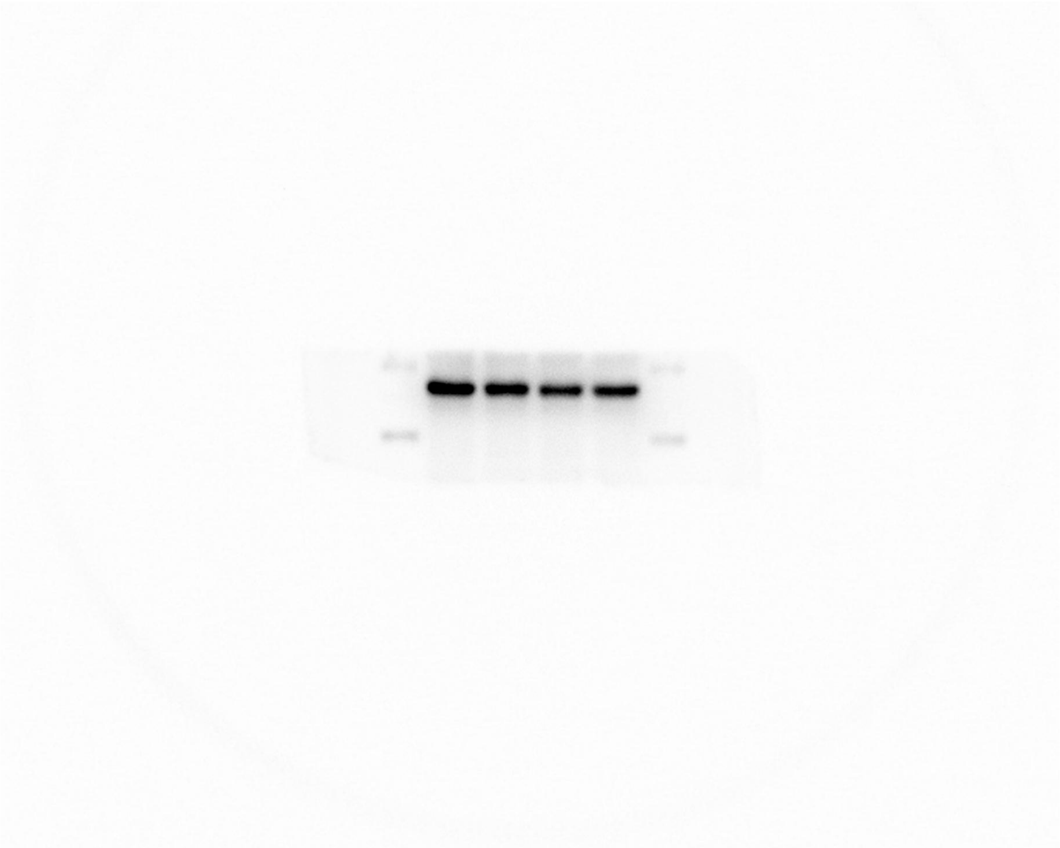

Figure 6G SUM159PT p-Akt

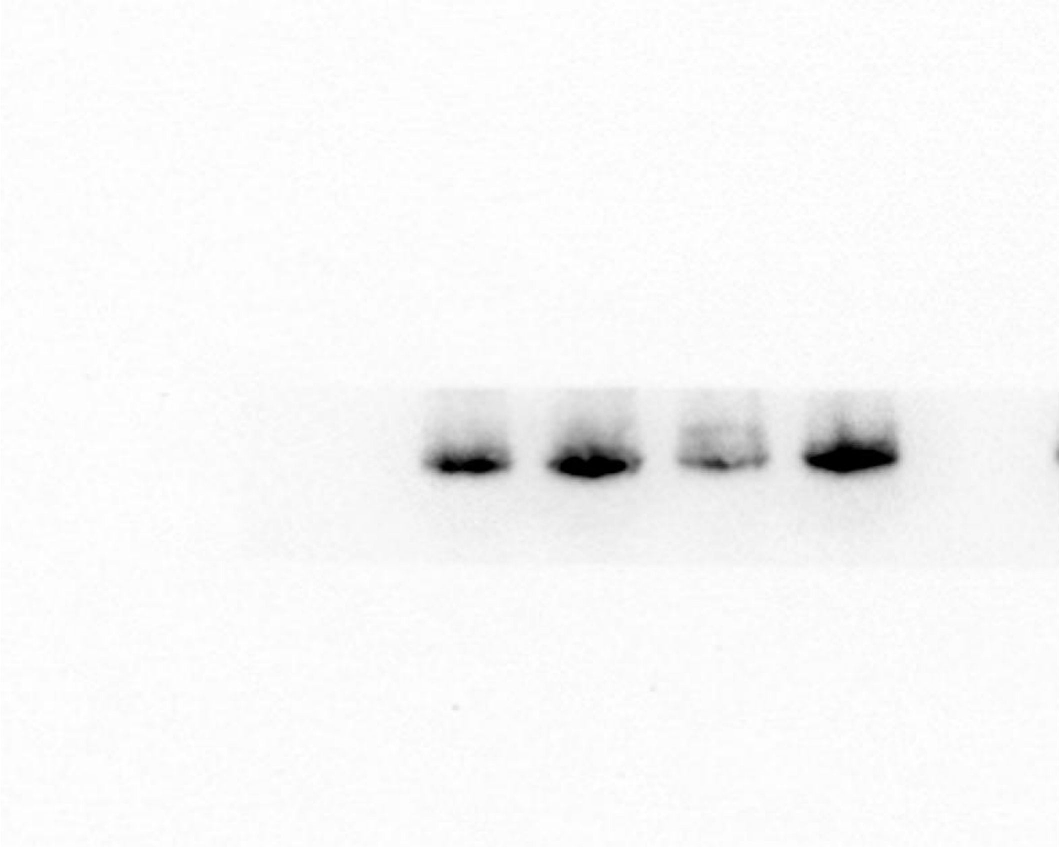

Figure 6G SUM159PT PI3K

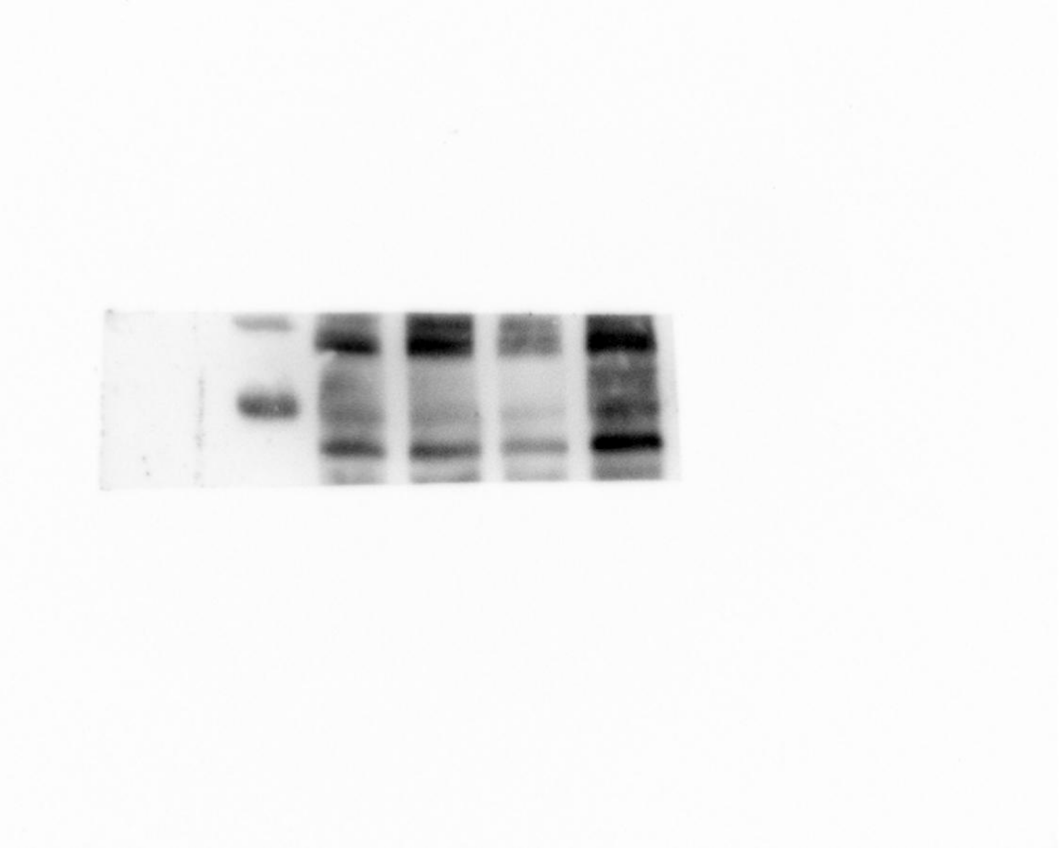

Figure 6G SUM159PT GAPDH

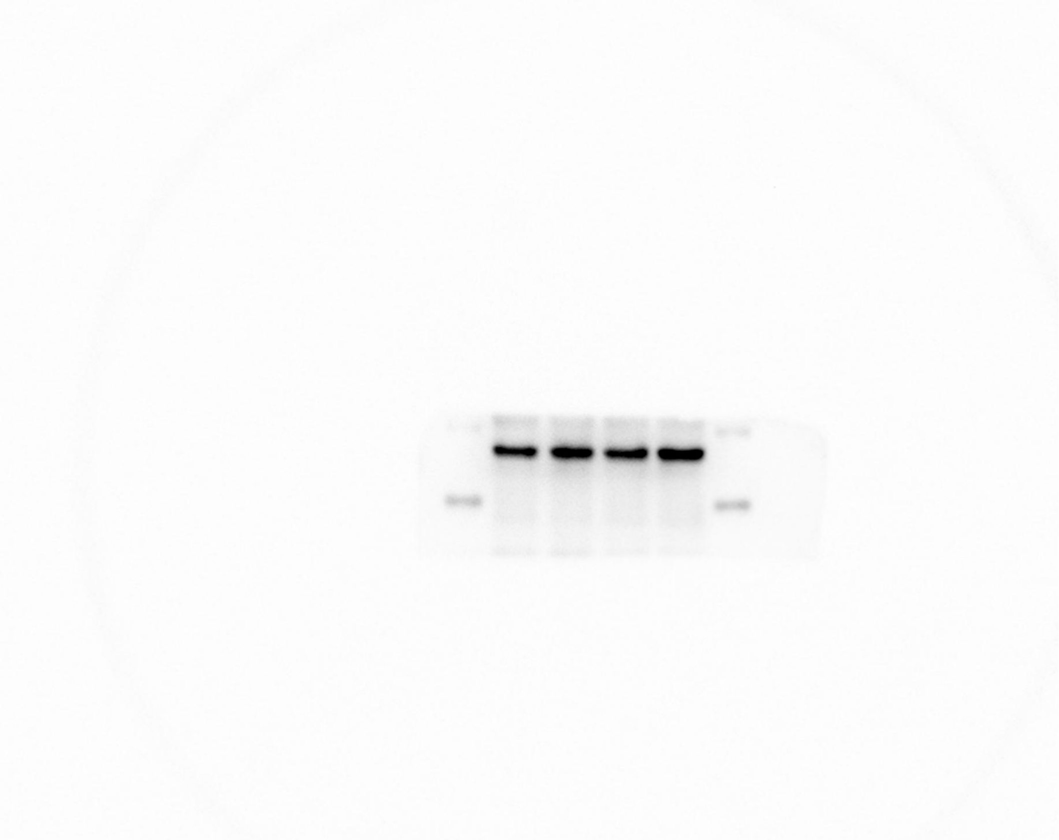

Figure 6G MDA-MB-231 Akt

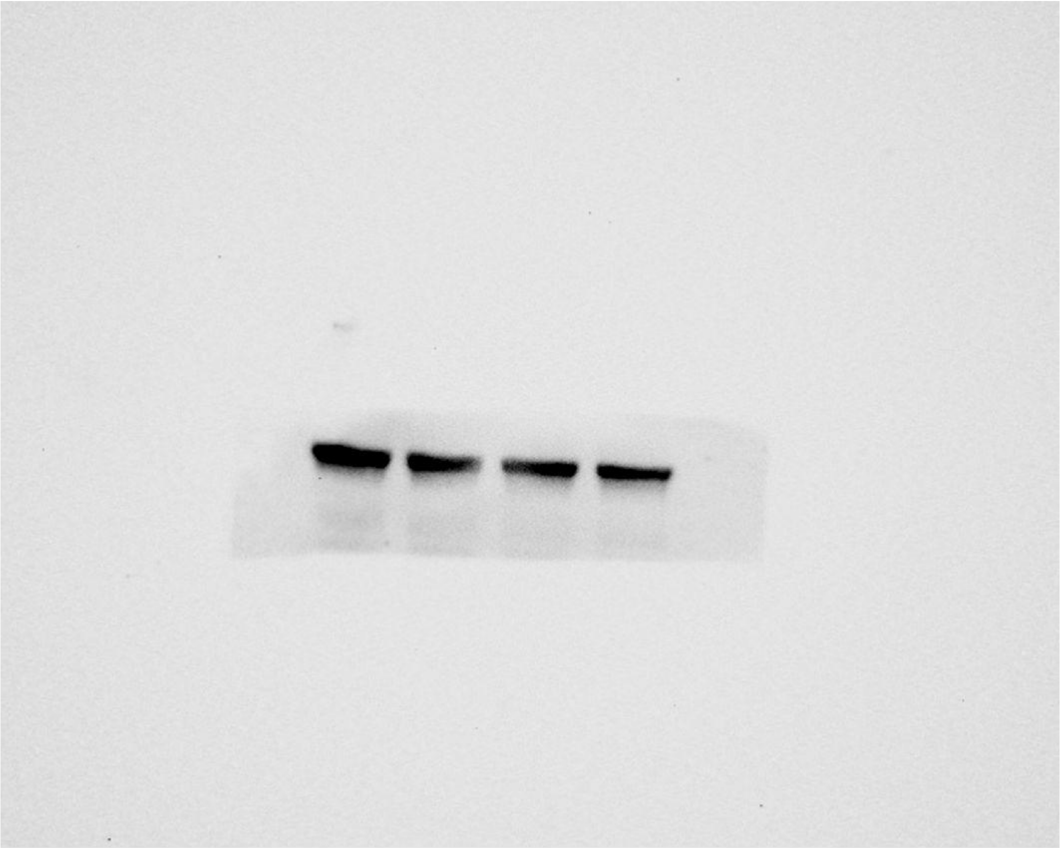

Figure 6G MDA-MB-231 p-Akt

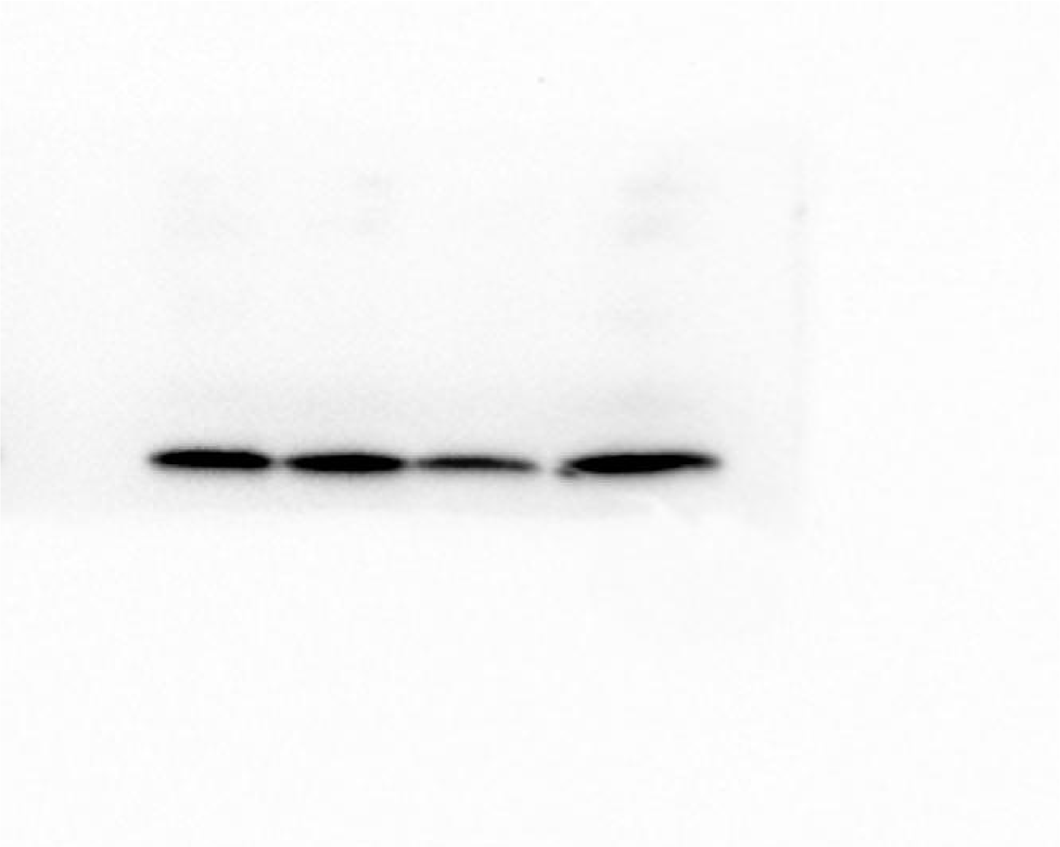

Figure 6G MDA-MB-231 PI3K

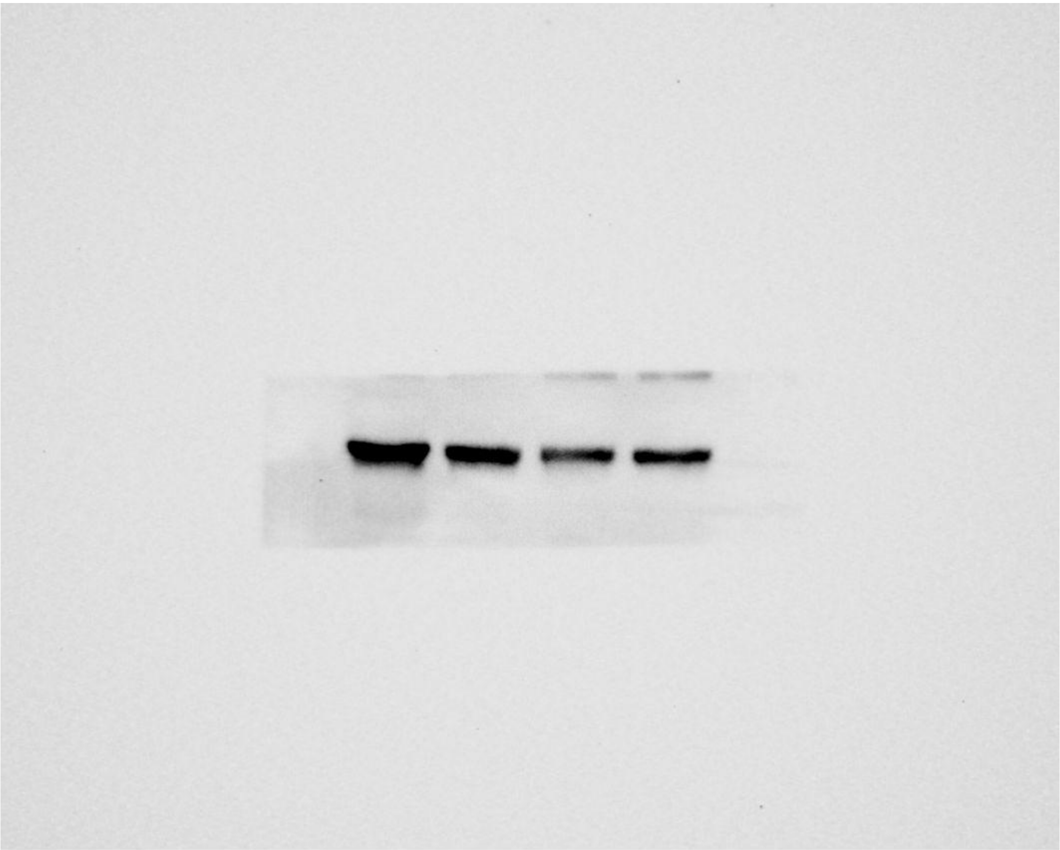

Figure 6G MDA-MB-231 GAPDH

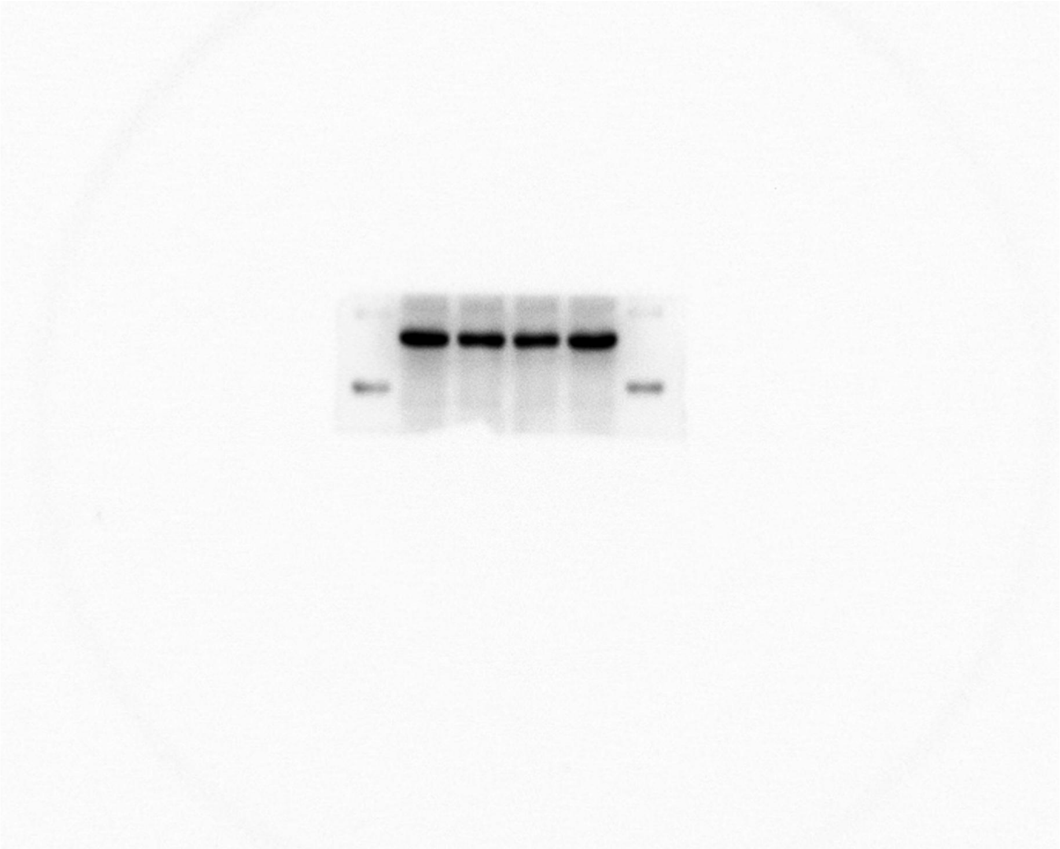

Figure 7E SUM159PT p53

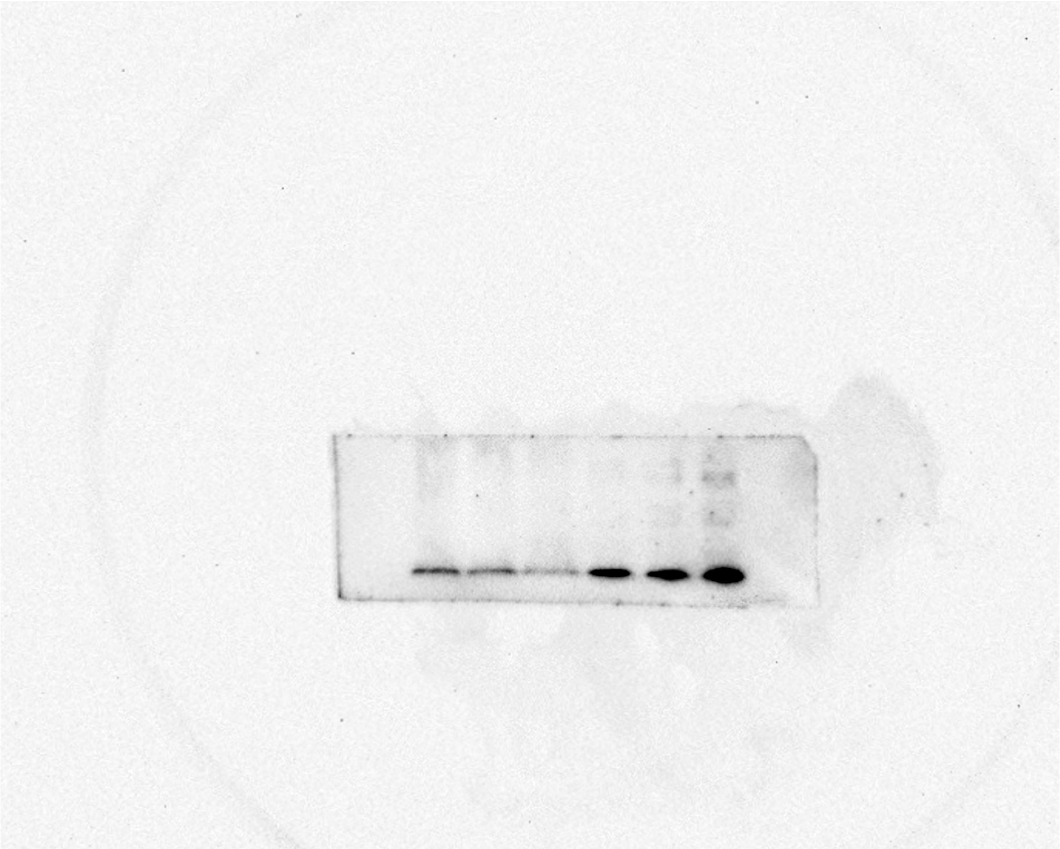

Figure 7E SUM159PT p21

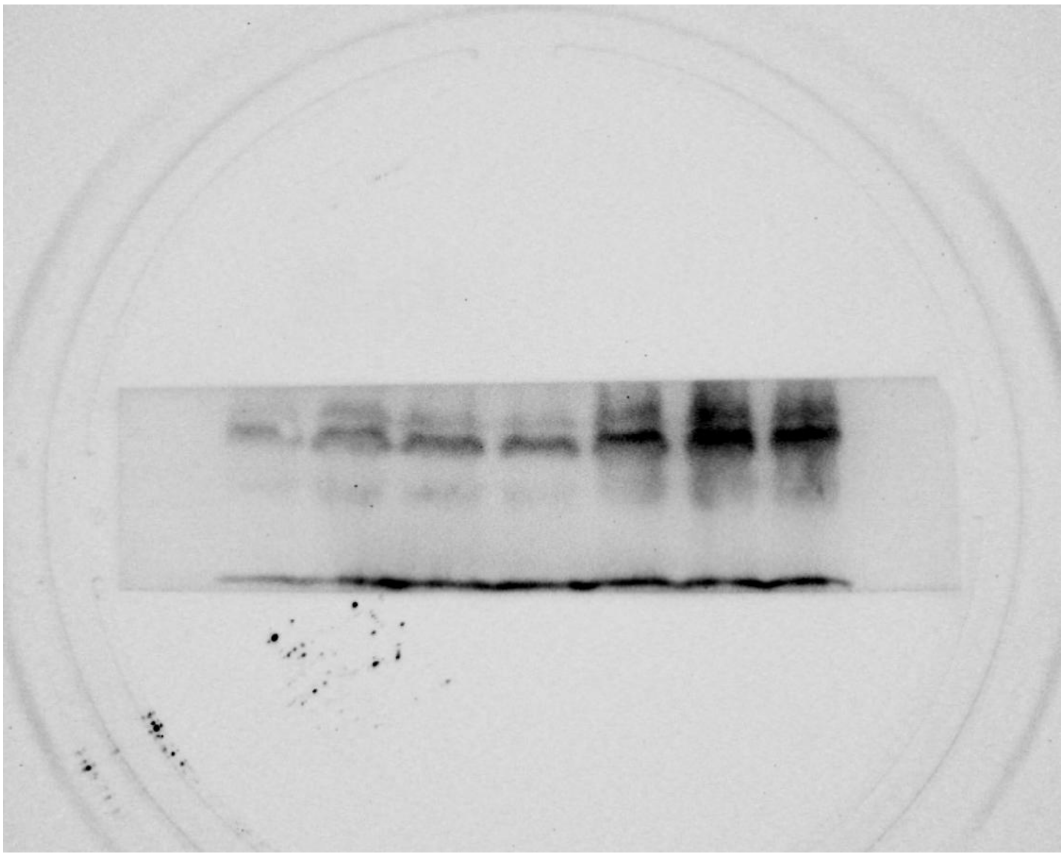

Figure 7E SUM159PT Akt

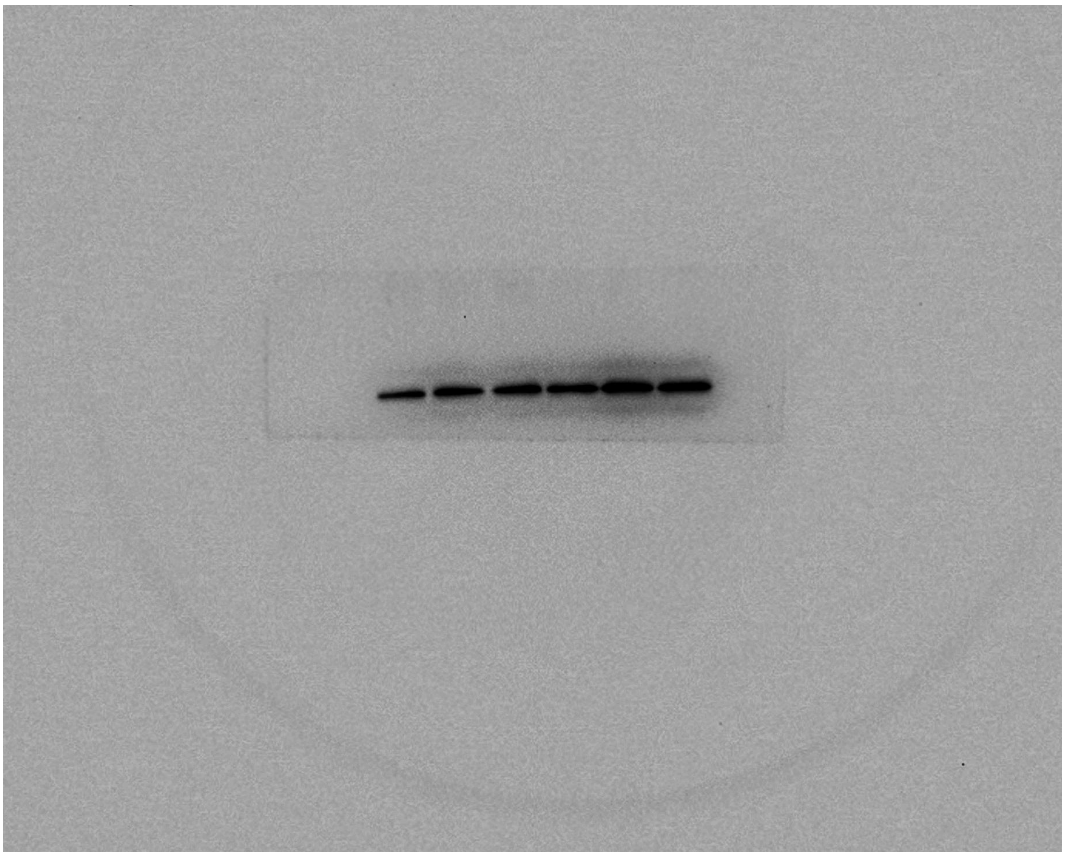

Figure 7E SUM159PT p-Akt

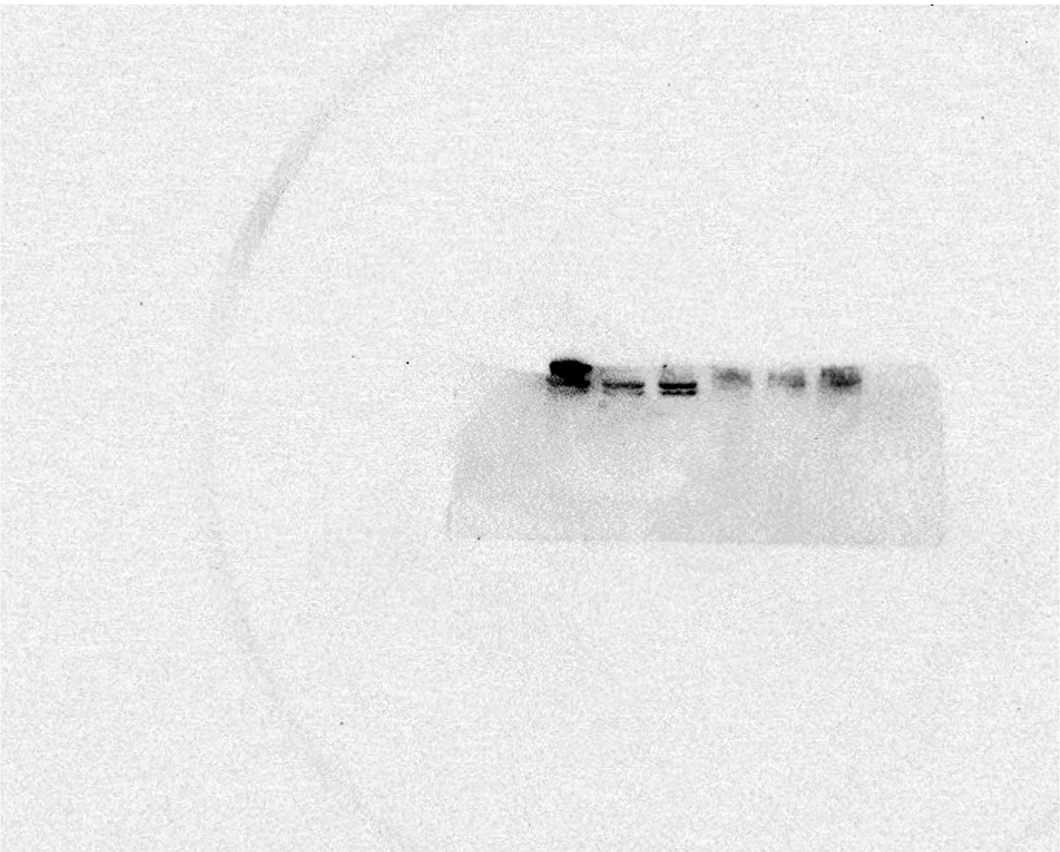

Figure 7E SUM159PT PI3K

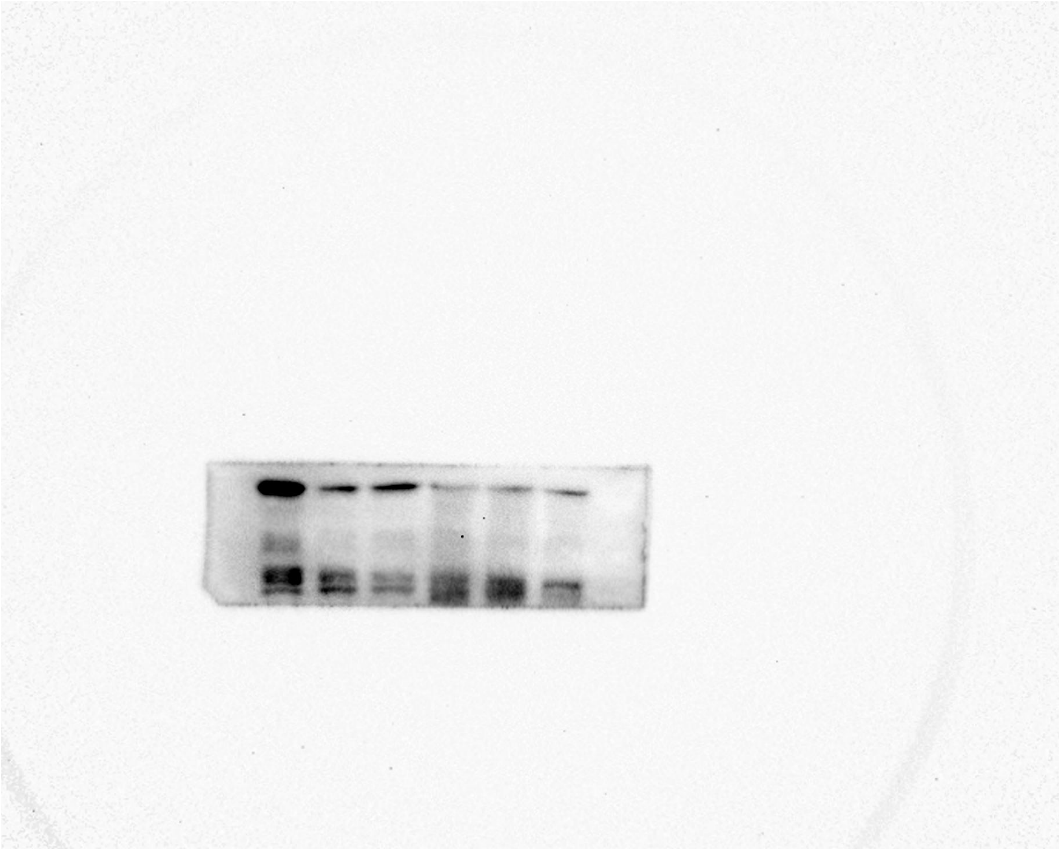

Figure 7E SUM159PT GAPDH

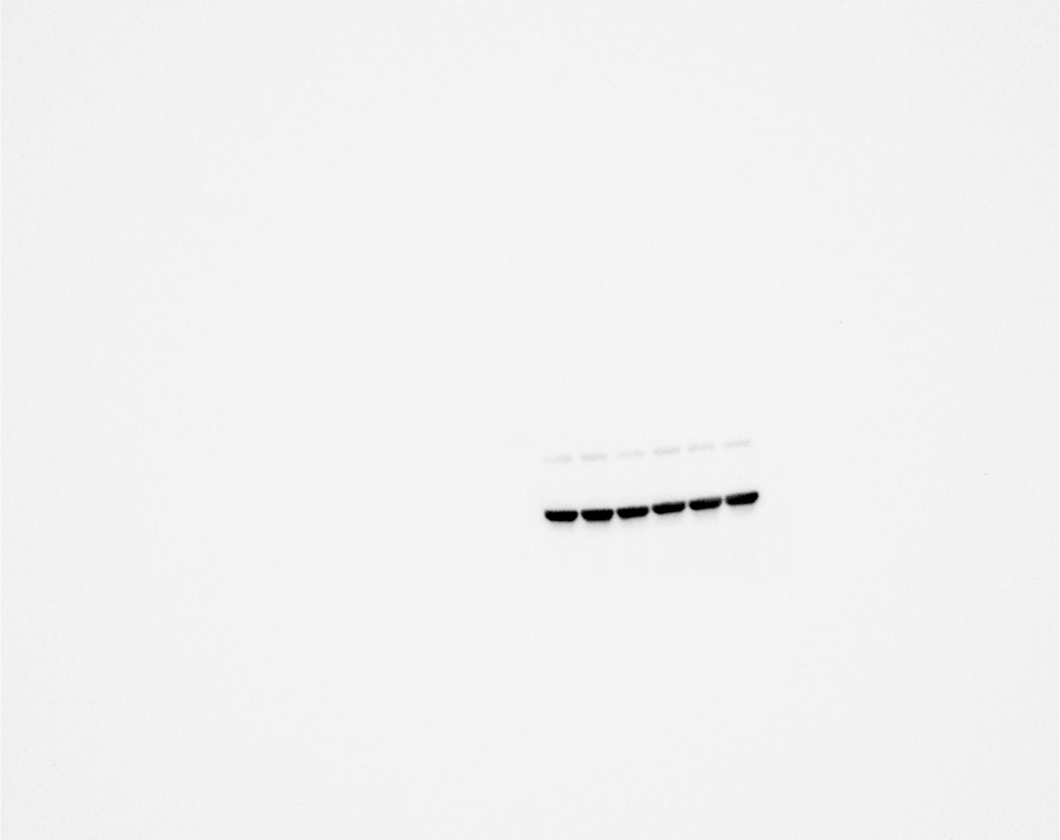

Figure 7E MDA-MB-231 p53

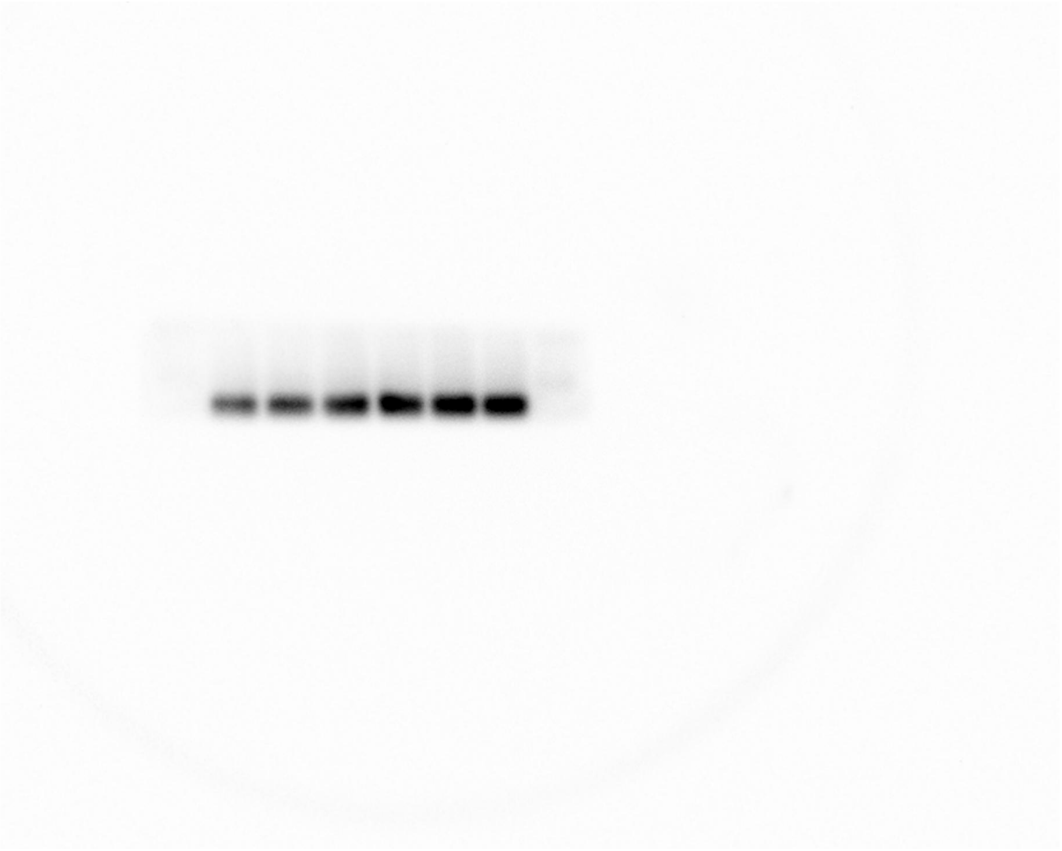

Figure 7E MDA-MB-231 p21

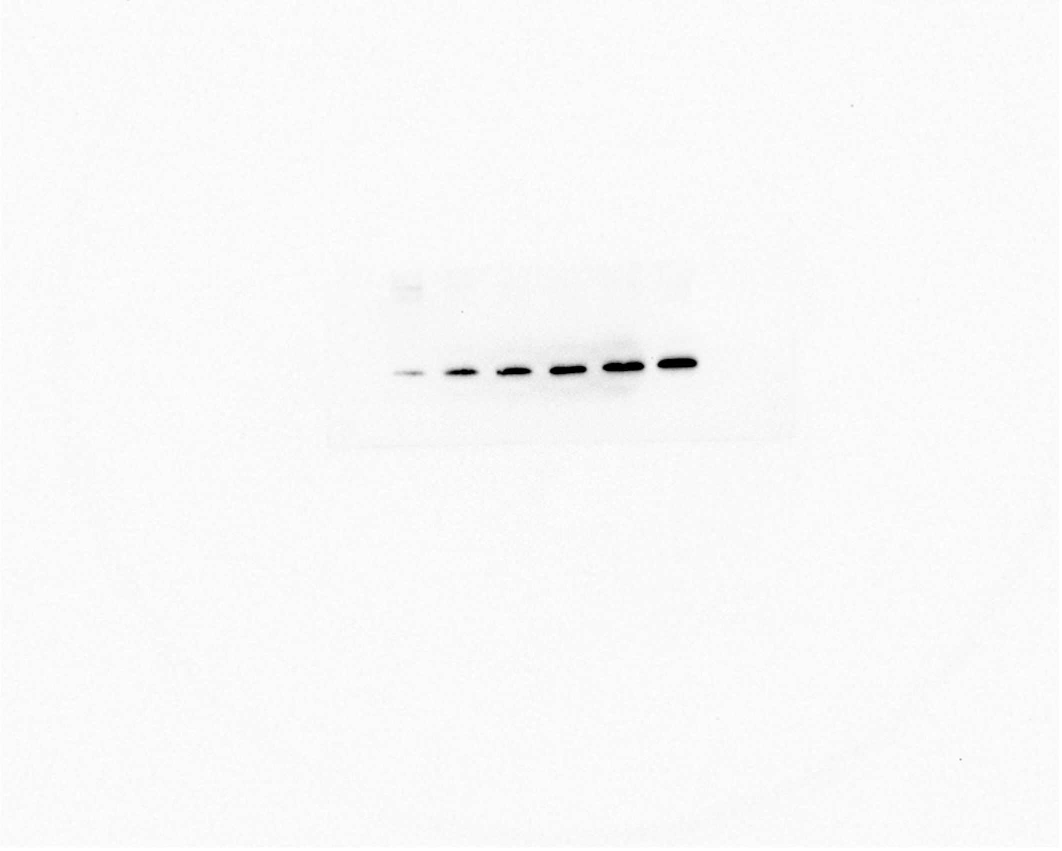

Figure 7E MDA-MB-231 Akt

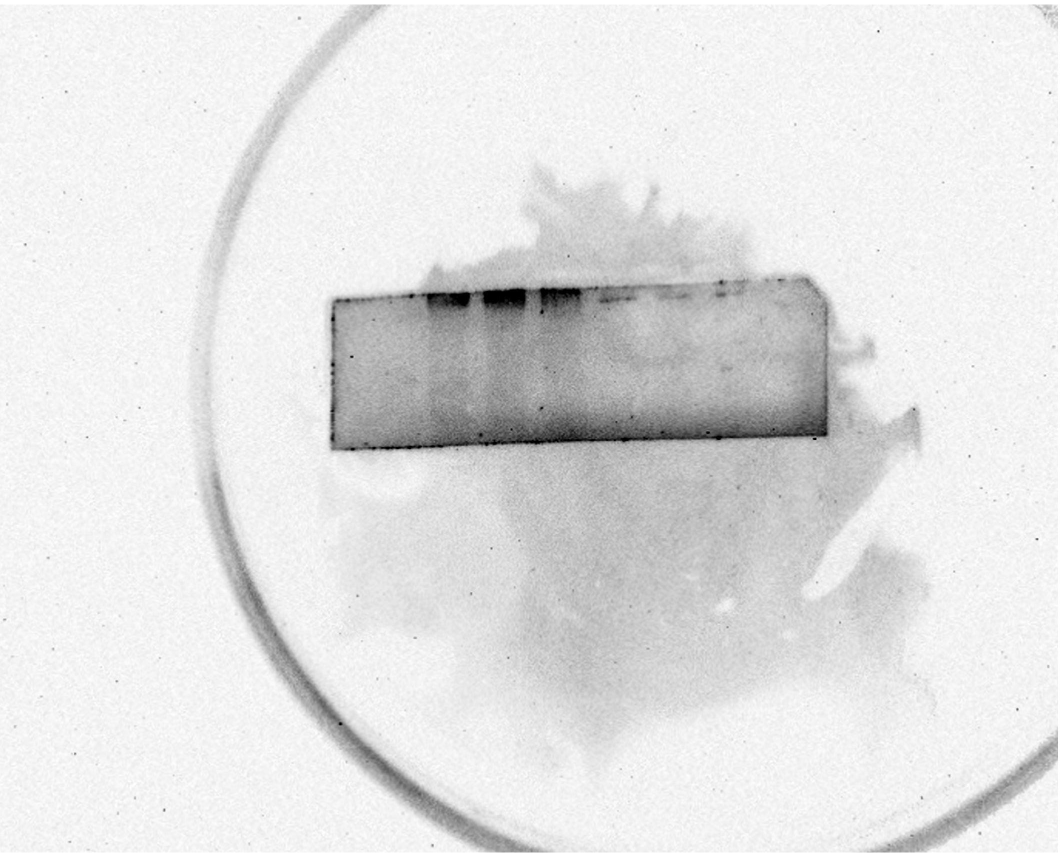

Figure 7E MDA-MB-231 PI3K

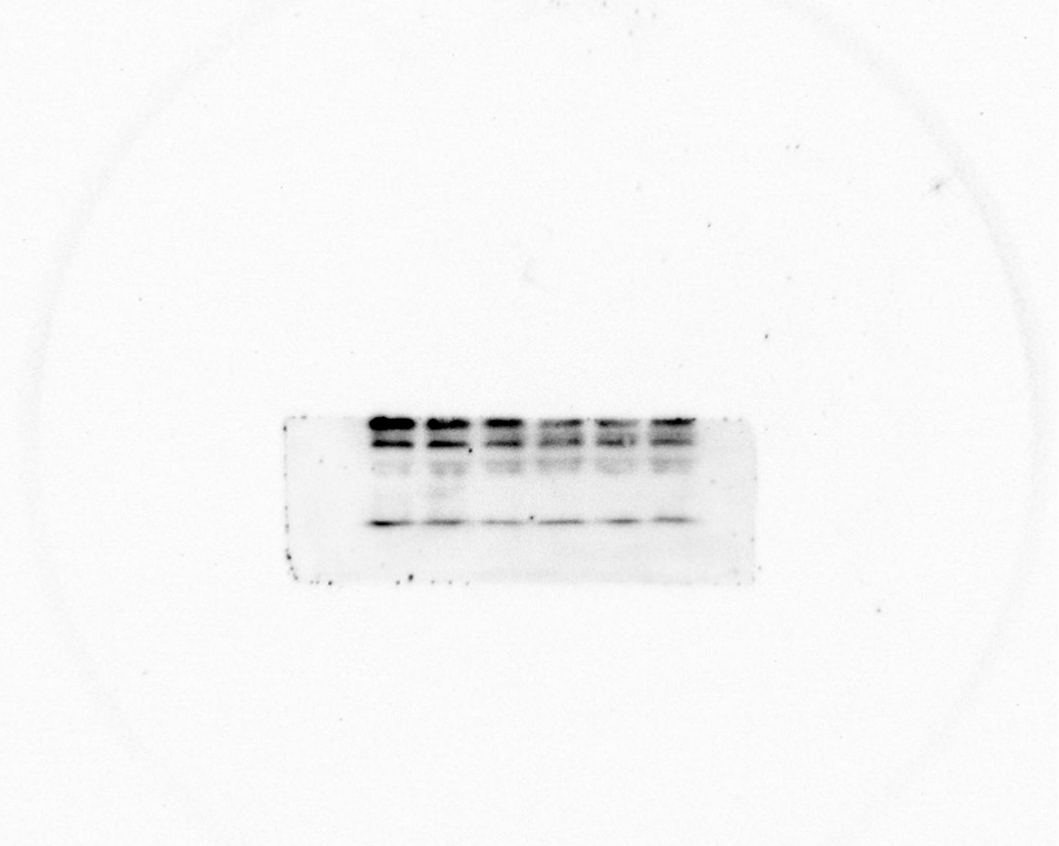

Figure 7E MDA-MB-231 GAPDH

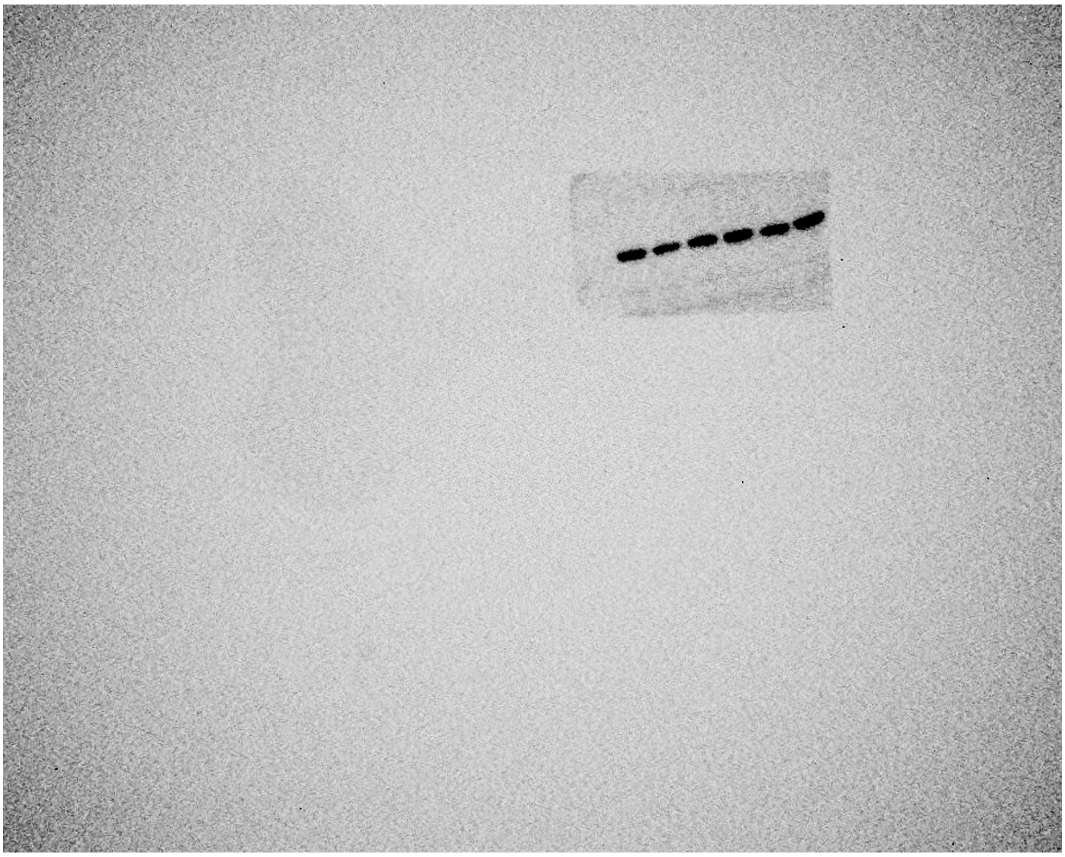

Supplement: Supplementary file 1 [file DataSheet4.pdf]
