# Supplementary material for: Saikosaponin A induces cellular senescence in triple-negative breast cancer by inhibiting the PI3K/Akt signalling pathway
Source: Front Pharmacol. 2025 Apr 25;16:1532579. doi: 10.3389/fphar.2025.1532579 (PMC12062077; doi:10.3389/fphar.2025.1532579)
Supplement: Supplementary file 2 [file Table1.docx]

**Supplementary table 1: Primer sequences are summarized**

| Genes | Primer sequences (5′→3′) |
| --- | --- |
| P21 | F: TGGAGACTCTCAGGGTCGAAA |
|  | R: GGCGTTTGGAGTGGTAGAAATC |
| P53 | F: GGACAGCCACGTCTGTGACTTG |
|  | R: CCAGTGGTTTCTTCTTTGGCTG |
| IL-1α | F: AGAGGAAGAAATCATCAAGC |
|  | R: TTATACTTTGATTGAGGGCG |
| IL-6 | F: GCAGAAAAGGCAAAGAATC |
|  | R: CTACATTTGCCGAAGAGC |
| GAPDH | F: GGAAGGTGAAGGTCGGAGTC |
|  | R: CGTTCTCAGCCTTGACGGT |
