# Supplementary material for: Saikosaponin A induces cellular senescence in triple-negative breast cancer by inhibiting the PI3K/Akt signalling pathway
Source: Front Pharmacol. 2025 Apr 25;16:1532579. doi: 10.3389/fphar.2025.1532579 (PMC12062077; doi:10.3389/fphar.2025.1532579)
Supplement: Supplementary file 3 [file DataSheet3.docx]

**Supplementary figure 3**


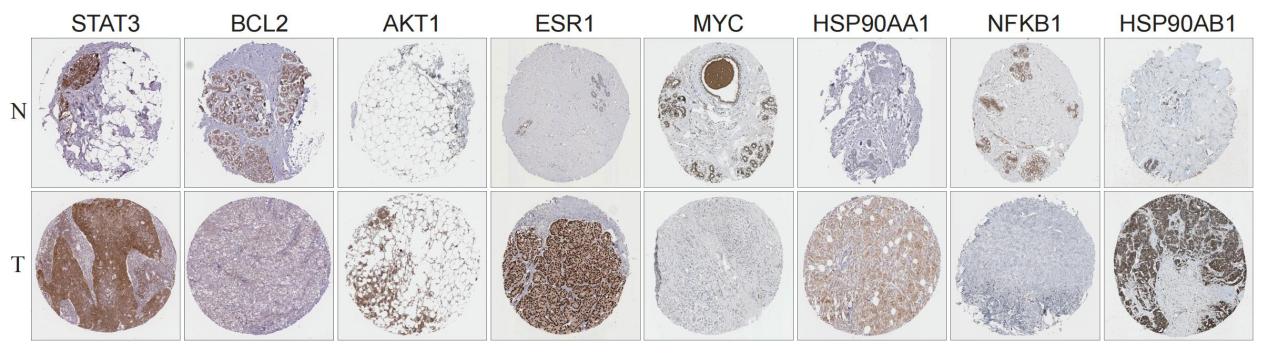


**Supplementary figure 3. The protein expression level of the 8 core targets in clinical specimens from the HPA database; N means normal tissue, T means tumor tissue.**
