# Supplementary material for: Saikosaponin A induces cellular senescence in triple-negative breast cancer by inhibiting the PI3K/Akt signalling pathway
Source: Front Pharmacol. 2025 Apr 25;16:1532579. doi: 10.3389/fphar.2025.1532579 (PMC12062077; doi:10.3389/fphar.2025.1532579)
Supplement: Supplementary file 4 [file DataSheet2.docx]

**Supplementary figure 2**


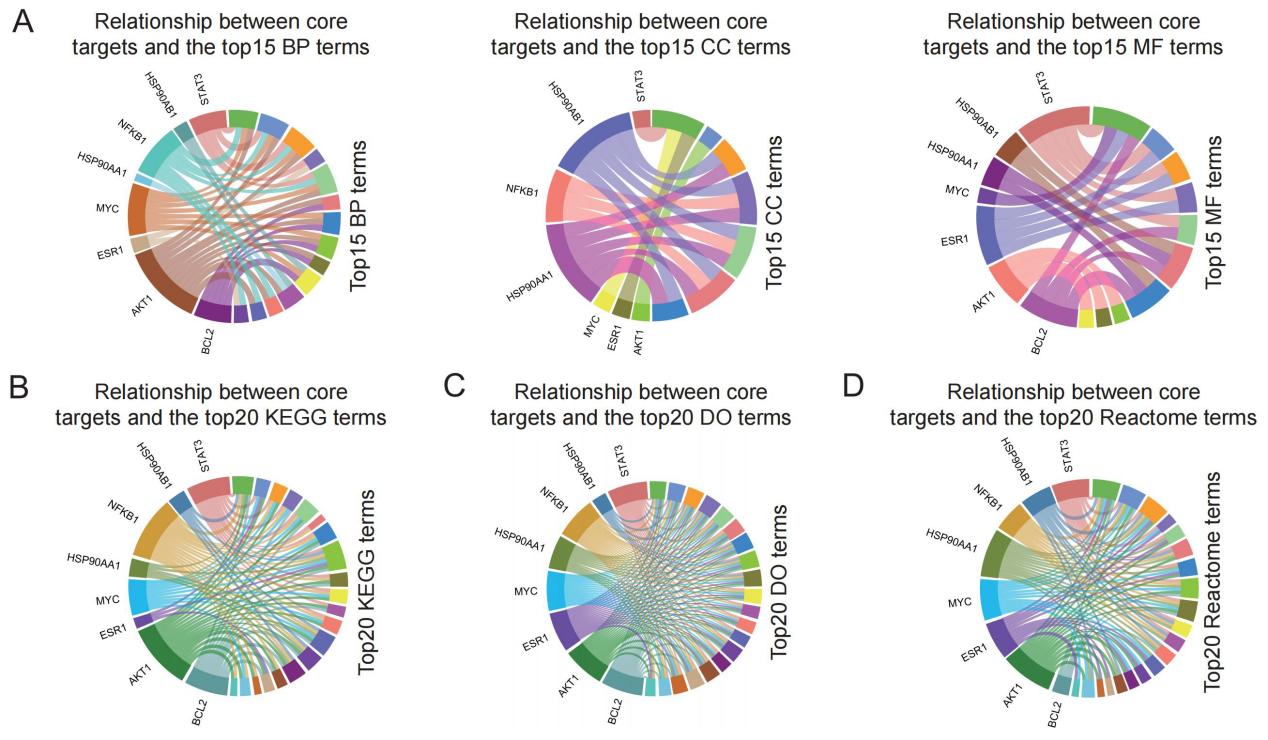


**Supplementary figure 2. Relationship of core targets with the top15 GO and top20 KEGG, DO, and** **Reactome pathways.** (A), (B), (C), and (D) Relationship of SSA core targets for TNBC with the top 15 GO, KEGG, DO and Reactome terms, respectively.
