# Supplementary material for: Saikosaponin A induces cellular senescence in triple-negative breast cancer by inhibiting the PI3K/Akt signalling pathway
Source: Front Pharmacol. 2025 Apr 25;16:1532579. doi: 10.3389/fphar.2025.1532579 (PMC12062077; doi:10.3389/fphar.2025.1532579)
Supplement: Supplementary file 5 [file DataSheet1.docx]

**Supplementary figure 1**


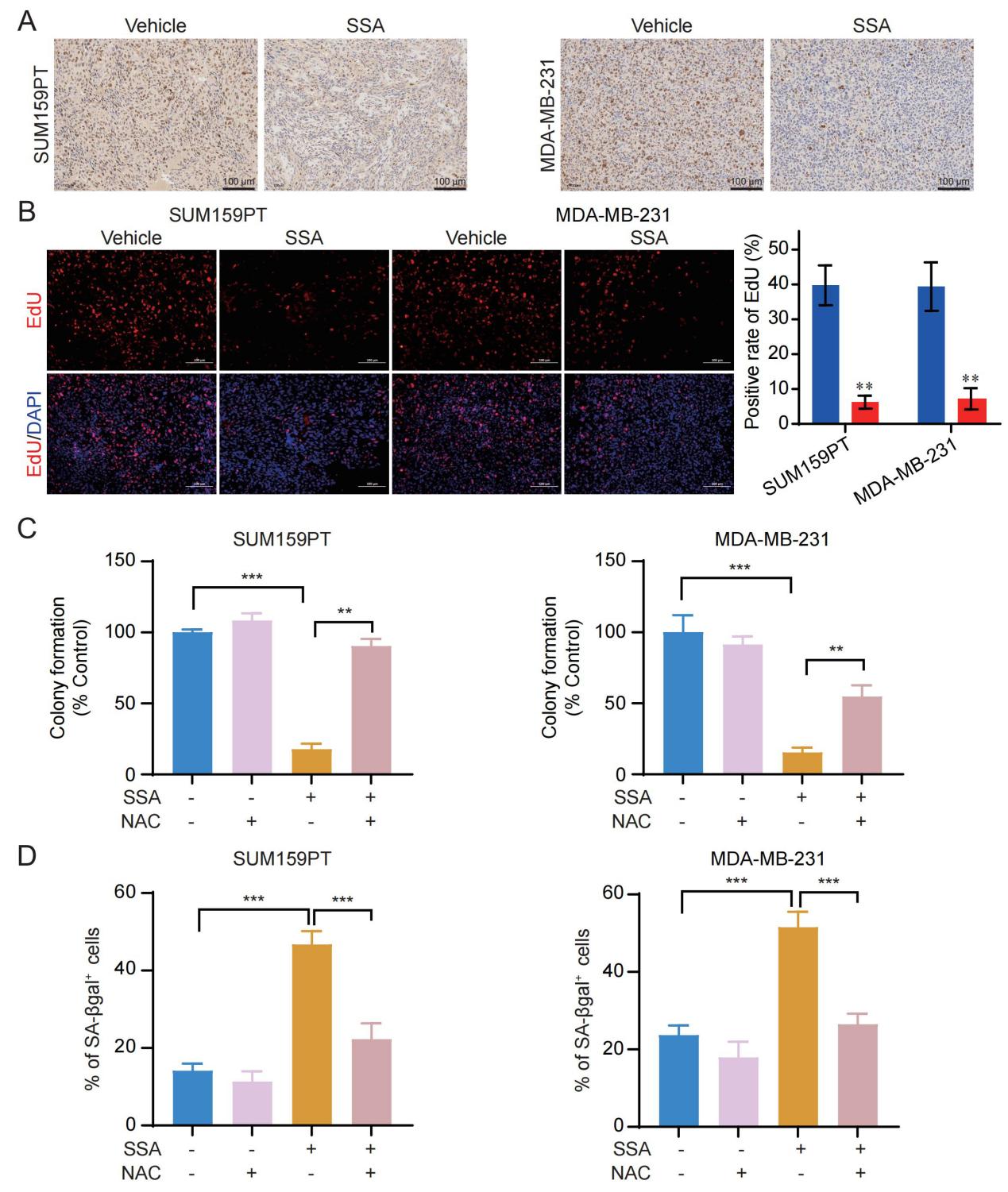


**Supplementary figure 1. NAC eliminated the effect of SSA on TNBC proliferation.** Ki67 immunohistochemistry (A) and the EdU labeling (B) results in the SSA-treatment and the vehicle groups were compared (n = 3). (C), and (D) Quantification of colony formation and SA-β-gal staining for SUM159PT and MDA-MB-231 cells treated with NAC (2 mM) in the presence or absence of 10 μM SSA for 48 h (n = 3). All the data are presented as the means ± SD. ^**^*P* < 0.01, ^***^*P* < 0.001.
